# Visualizing Phytochemical-Protein Interaction Networks: *Momordica charantia* and Cancer

**DOI:** 10.3389/fbinf.2021.768886

**Published:** 2021-12-13

**Authors:** Yumi L. Briones, Alexander T. Young, Fabian M. Dayrit, Armando Jerome De Jesus, Nina Rosario L. Rojas

**Affiliations:** ^1^ Department of Chemistry, Ateneo de Manila University, Quezon City, Philippines; ^2^ Institute of Environmental Science & Meteorology, College of Science, University of the Philippines Diliman, Quezon City, Philippines

**Keywords:** network visualization, network pharmacology, reverse screening, medicinal plants, phytochemicals, Momordica charantia (bitter gourd), colorectal cancer

## Abstract

The *in silico* study of medicinal plants is a rapidly growing field. Techniques such as reverse screening and network pharmacology are used to study the complex cellular action of medicinal plants against disease. However, it is difficult to produce a meaningful visualization of phytochemical-protein interactions (PCPIs) in the cell. This study introduces a novel workflow combining various tools to visualize a PCPI network for a medicinal plant against a disease. The five steps are 1) phytochemical compilation, 2) reverse screening, 3) network building, 4) network visualization, and 5) evaluation. The output is a PCPI network that encodes multiple dimensions of information, including subcellular location, phytochemical class, pharmacokinetic data, and prediction probability. As a proof of concept, we built a PCPI network for bitter gourd (*Momordica charantia* L.) against colorectal cancer. The network and workflow are available at https://yumibriones.github.io/network/. The PCPI network highlights high-confidence interactions for further *in vitro* or *in vivo* study. The overall workflow is broadly transferable and can be used to visualize the action of other medicinal plants or small molecules against other diseases.

## 1 Introduction

Medicinal plants have been consumed to fight disease since ancient times (Petrovska, 2012). However, even in the modern age, their complex cellular action is not fully understood. Unlike magic bullets that selectively target a given protein, phytochemicals in medicinal plants act on multiple protein targets to restore the overall equilibrium of the cell (Ding et al., 2009). While *in vitro* and *in vivo* methods are often used to study the therapeutic effects of medicinal plants, there is limited experimental data on phytochemical-protein interactions (PCPIs) (Huang et al., 2018). Recently there has been increasing use of *in silico* methods such as reverse screening and network pharmacology in natural products research, as these are well-suited for studying the multi-targeted action of medicinal plants (Chandran et al., 2017).

Reverse screening uses experimentally validated PCPIs to make novel predictions. While conventional screening starts with a target protein and searches for compounds targeting it, reverse screening starts with the compounds (e.g. phytochemicals) and looks for proteins targeted by these compounds (Huang et al., 2018). The ability of reverse screening to predict PCPIs makes it useful for a network pharmacology approach where phytochemicals and proteins are analyzed as nodes in an interaction network. Of all existing reverse screening tools we are aware of, only one provides a network visualization: Bioinformatics Analysis Tool for Molecular mechANism of Traditional Chinese Medicine (BATMAN-TCM) (Liu et al., 2016). The network shows predicted interactions between phytochemicals, protein targets, and enriched pathways and diseases. However, this does not provide a complete picture of the action of a medicinal plant against a specific disease, which is often the goal of natural products research. It would be useful to see protein-protein interactions (PPIs) between targets to evaluate downstream effects. The network can be better organized by sorting nodes into subcellular compartments. To assess whether phytochemicals can reach these compartments, pharmacokinetic properties are needed. There are existing tools for each of these purposes, but they are all separately found.

Natural products research would greatly benefit from a streamlined workflow that results in a strong (PCPI) network visualization. Thus, we developed a novel workflow combining existing tools to predict and visualize the cellular action of a medicinal plant against a disease. The five-step pipeline consists of 1) phytochemical compilation, 2) reverse screening, 3) network building, 4) network visualization, and 5) evaluation. This outputs a PCPI network that encodes multiple dimensions of information including PPIs, subcellular location, phytochemical class, and pharmacokinetic properties. This makes it easier to determine which predicted PCPIs merit further *in vitro* and *in vivo* study.

As a proof of concept, we applied the workflow to *Momordica charantia* L. (bitter gourd) against colorectal cancer. Bitter gourd has shown anticancer activity *in vitro* and *in vivo* but has not been thoroughly investigated *in silico* (Raina et al., 2016). Meanwhile, colorectal cancer is a disease known to be highly influenced by diet (Dray et al., 2003). We evaluated select PCPIs by molecular docking and identified high-confidence predictions for further study. Our website (https://yumibriones.github.io/network/) contains the PCPI network we generated and a diagram of the workflow with links to all resources used. With this study, we aim to improve the efficiency of natural products research by using readily available tools to produce insightful network visualizations.

## 2 Methods

### 2.1 General Workflow

The general workflow consists of five main steps:

1) Phytochemical compilation: A medicinal plant is chosen and searched in a phytochemical database and literature to obtain a “Phytochemical list.”
2) Reverse screening: The “Phytochemical list” is entered in a reverse screening program to obtain a “Complete PCPIs” list.
3) Network building: Protein targets from the “Complete PCPIs” list are run through pathway enrichment after which a disease is chosen. The “Disease-specific PCPIs” are merged with the existing PPI network Signaling Network Open Resource (SIGNOR) 2.0 to output a “PCPI-SIGNOR disease network.” Information on phytochemical class, pharmacokinetic properties, subcellular location and protein function are added using various resources.
4) Network visualization: The “Annotated PCPI-SIGNOR disease network” is visualized using Cytoscape and arranged by subcellular location using the plug-in boundaryLayout. Phytochemical and protein attributes are visualized.
5) Evaluation: The “PCPI-SIGNOR disease network visualization” is analyzed and notable PCPIs are evaluated *in silico*, *in vitro*, or *in vivo.*

**FIGURE 1:**
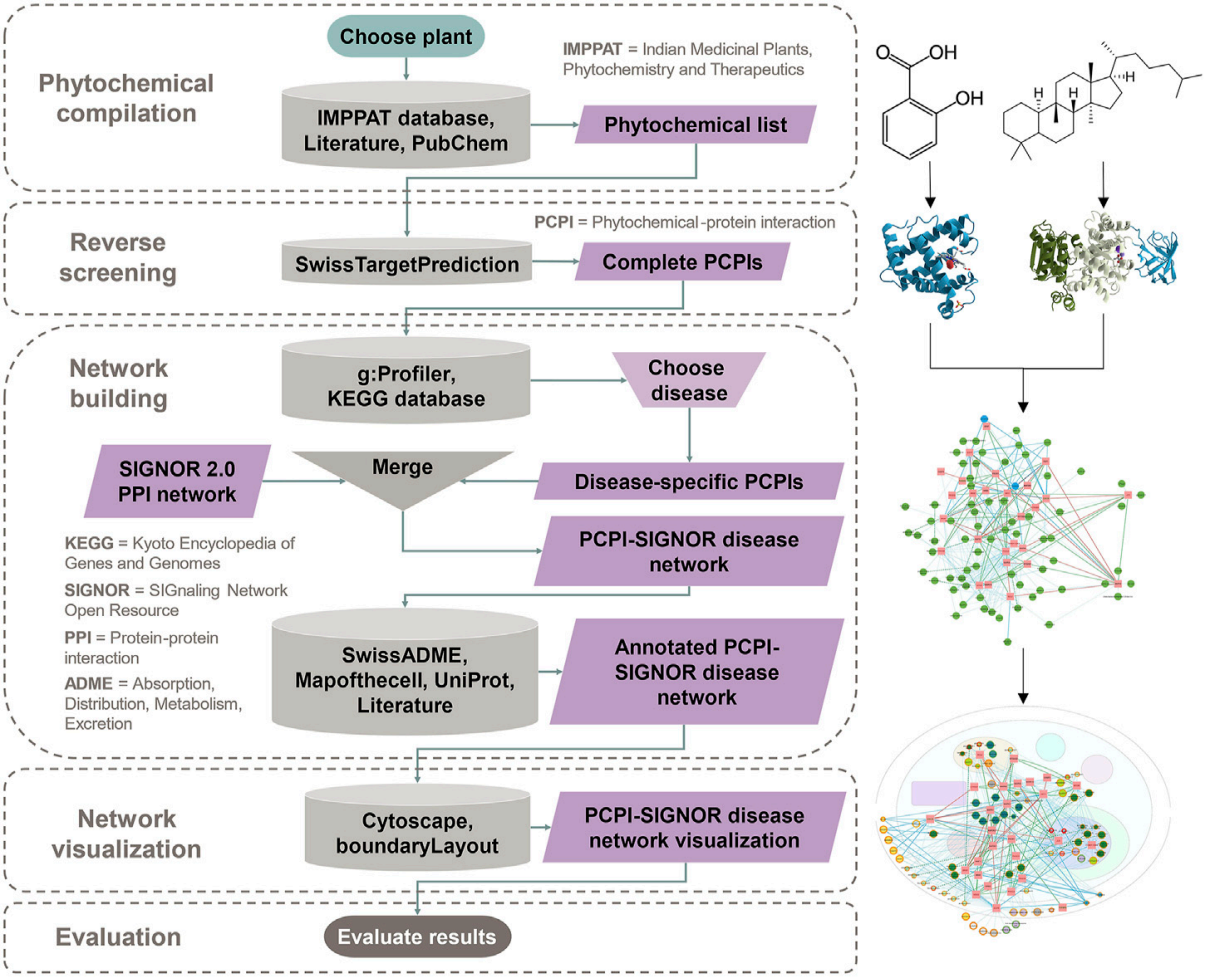
The workflow begins with phytochemical compilation for the chosen plant. This is followed by reverse screening to predict PCPIs. Network building filters PCPIs to the chosen disease, combines these with PPIs, and annotates the network with phytochemical and protein attributes. The annotated network is visualized with Cytoscape. Evaluation is done through any *in silico*, *in vitro*, or *in vivo* method. This flowchart and links to resources can be found at https://yumibriones.github.io/network/workflow.html.

Figure 1 is a detailed diagram of the workflow showing inputs and outputs of each step and all resources used in the study.

The following sections provide more detail for each step, including brief backgrounds on each resource used.

### 2.2 Phytochemical Compilation

After choosing a medicinal plant to investigate, the plant is entered into the Indian Medicinal Plants, Phytochemistry And Therapeutics (IMPPAT) (https://cb.imsc.res.in/imppat) database. IMPPAT contains phytochemical-plant associations mined from medicinal plant books, phytochemical databases, and PubMed abstracts (Mohanraj et al., 2018). Phytochemicals may also be determined from the literature. Positive and negative control molecules may be selected. If drugs are selected as controls, their interactions are referred to as drug-protein interactions (DPIs). The Simplified Molecular Input Line Entry System (SMILES) of all molecules are obtained from PubChem (Kim et al., 2019). Phytochemicals are sorted by class according to Medical Subject Headings (MeSH) Tree (U.S. National Library of Medicine, 2021) or Chemical Entities of Biological Interest (ChEBI) ontology (Hastings et al., 2016).

To simulate metabolism, glycosides (molecules bonded to sugar units) are manually hydrolyzed with molecular editing software such as ChemSketch, developed by Advanced Chemistry Development, Inc. (ACD/Labs). Both glycosides and aglycones (the non-sugar unit) are kept in the list of phytochemicals, combining any duplicate structures into a single entry. The complete resulting list is the “Phytochemical list” from Figure 1.

### 2.3 Reverse Screening

Reverse screening is done with SwissTargetPrediction (http://www.swisstargetprediction.ch), a shape screening software that uses ligand-protein binding data from ChEMBL version 23 (Mendez et al., 2019). When a query molecule is entered, SwissTargetPrediction calculates 2D and 3D similarity scores with ligands in the database. Both scores are combined to obtain the probability that the query molecule shares the same protein target as the matched ligands (Daina et al., 2019). If the query molecule is already listed in the ChEMBL database, SwissTargetPrediction assigns a prediction probability of 1.

Molecules in the “Phytochemical list” from the previous step are entered into SwissTargetPrediction using the SMILES, with *Homo sapiens* as the selected organism. The output is a list of predicted protein targets and probability scores for the query molecule which can be downloaded as a CSV file. Only results with probabilities greater than zero are considered. The combined list of predictions for all molecules in the “Phytochemical list” is the “Complete PCPIs” output.

### 2.4 Network Building

Network building consists of four steps: 1) pathway enrichment, 2) addition of PPIs and glycoside-aglycone relationships, 3) assignment of subcellular locations, and 4) pharmacokinetic analysis of phytochemicals.

#### 2.4.1 Pathway Enrichment

The program g:Profiler (https://biit.cs.ut.ee/gprofiler/gost) (Reimand et al., 2007) is used to identify statistically overrepresented pathways in the set of predicted protein targets from the “Complete PCPIs” list. The protein names are entered as a query, and the search is carried out with *Homo sapiens* as the selected organism, a 0.05 significance threshold, and Kyoto Encyclopedia of Genes and Genomes (KEGG) (Kanehisa and Goto, 2000) as the reference database. Results are downloaded as a CSV file which lists all enriched pathways and intersected proteins per pathway. From the file, the disease of interest is located. The intersected proteins under the disease are used to filter the “Complete PCPIs” list to only the “Disease-specific PCPIs.”

#### 2.4.2 Addition of PPIs and Glycoside-aglycone Relationships

The SIGNOR 2.0 database is used as a source of PPIs. SIGNOR 2.0 is a biological network of literature-based causal interactions between proteins. The entire network is directed from source to target node (Licata et al., 2020). The full *Homo sapiens* database was downloaded on September 28, 2020. Tableau Prep is used to combine the “Disease-specific PCPIs” with PPIs from SIGNOR 2.0 using the disease-specific protein targets as a join clause. Glycoside-aglycone relationships are added to the network as interactions directed from the parent glycoside to child aglycone. The resulting file is the “PCPI-SIGNOR disease network” (Figure 1). In this file, all source nodes are labelled “Entity A” while all target nodes are labelled “Entity B.”

#### 2.4.3 Assignment of Subcellular Locations

All proteins in the “PCPI-SIGNOR disease network” are assigned a subcellular location using an interactive database of the HeLa spatial proteome developed by Itzhak et al. (2016) (http://mapofthecell.biochem.mpg.de/). The database is a downloadable Excel file that reports the most probable cellular location of a protein based on fractionation and mass spectrometry experiments. When protein names are entered into the file, the corresponding subcellular locations will appear.

UniProt is used for proteins not in the HeLa database. Protein entries in UniProt contain a “Subcellular location” section based on expert annotations (The UniProt Consortium, 2021). The Gene Ontology (GO) tool is not chosen for this step, as it often outputs a long list of all recorded links between a protein and cellular component with no way to narrow down options (Hill et al., 2008).

Subcellular locations of phytochemicals and controls are assigned in this order of priority:

1) ligands with protein targets in the nucleus were placed in the nucleus;
2) ligands with protein targets in the mitochondrion were placed in the mitochondrion;
3) ligands with protein targets in the plasma membrane were placed in the plasma membrane; and
4) ligands with protein targets in the cytoplasm were placed in the cytoplasm.

In the “PCPI-SIGNOR disease network” file, the subcellular locations of “Entity A” and “Entity B” are entered into separate columns labelled “Location A” and “Location B” respectively. This results in an “Annotated PCPI-SIGNOR disease network” file.

#### 2.4.4 Pharmacokinetic Analysis of Phytochemicals

SwissADME (http://www.swissadme.ch/) assesses physicochemical and pharmacokinetic parameters of input molecules (Daina et al., 2017). All phytochemicals and controls included in the “Disease-specific PCPIs” are entered into SwissADME using their name and SMILES. The results are a list of pharmacokinetic data for each molecule. The results are downloaded as a CSV file and the following attributes are noted: Abbott bioavailability score, gastrointestinal (GI) absorption (for orally ingested medicinal plants), and lipophilicity using the partition coefficient   log  *p* (Eq. 1). A more lipophilic compound would have a higher log  *p* value.

$$\log P=\log_{10}\frac{\left[concentration\;of\;solute\;in\;octanol\right]}{\left[concentration\;of\;solute\;in\;water\right]}$$ (1)

Each pharmacokinetic parameter is entered as its own column in the “Annotated PCPI-SIGNOR disease network” file. Columns modifying “Entity A” or “Entity B” are ended with “A” or “B” respectively (e.g. “Bioavailability A”).

### 2.5 Network Visualization

The “Annotated PCPI-SIGNOR disease network” Excel file is loaded into Cytoscape 3.6.0 (Shannon et al., 2003). All duplicate edges and self-loops are removed.

For edges, these parameters are followed:

1) edge thickness is mapped to the SwissTargetPrediction probability score (thicker edges = more probable); and
2) edge color is mapped to interaction type (predicted PCPI or DPI = blue, PPI upregulation = green, PPI downregulation = red, glycoside-aglycone relation = dark green dashed line).

For ligand nodes, these parameters are followed:

1) node shape is set to circle;
2) node transparency is mapped to log  *P* value (lower log  *P* = more transparent, higher log  *P* = more opaque);
3) node size is mapped to GI absorption (high absorption = large, low absorption = small);
4) node border color is mapped to Abbott bioavailability score (lowest scores in red, highest scores in green); and
5) node color was mapped to ligand class.

For protein nodes, these parameters are followed:

1) node shape is set to square;
2) node color is set to pink; and
3) label color is mapped to protein function (red = oncogene protein, green = tumor suppressor, black = other protein).

Nodes are automatically organized into a cell template based on the assigned cellular location using the Cytoscape plug-in boundaryLayout, developed by University of California San Francisco’s Resource for Biocomputing, Visualization, and Informatics (UCSF RBVI).

**FIGURE 2:**
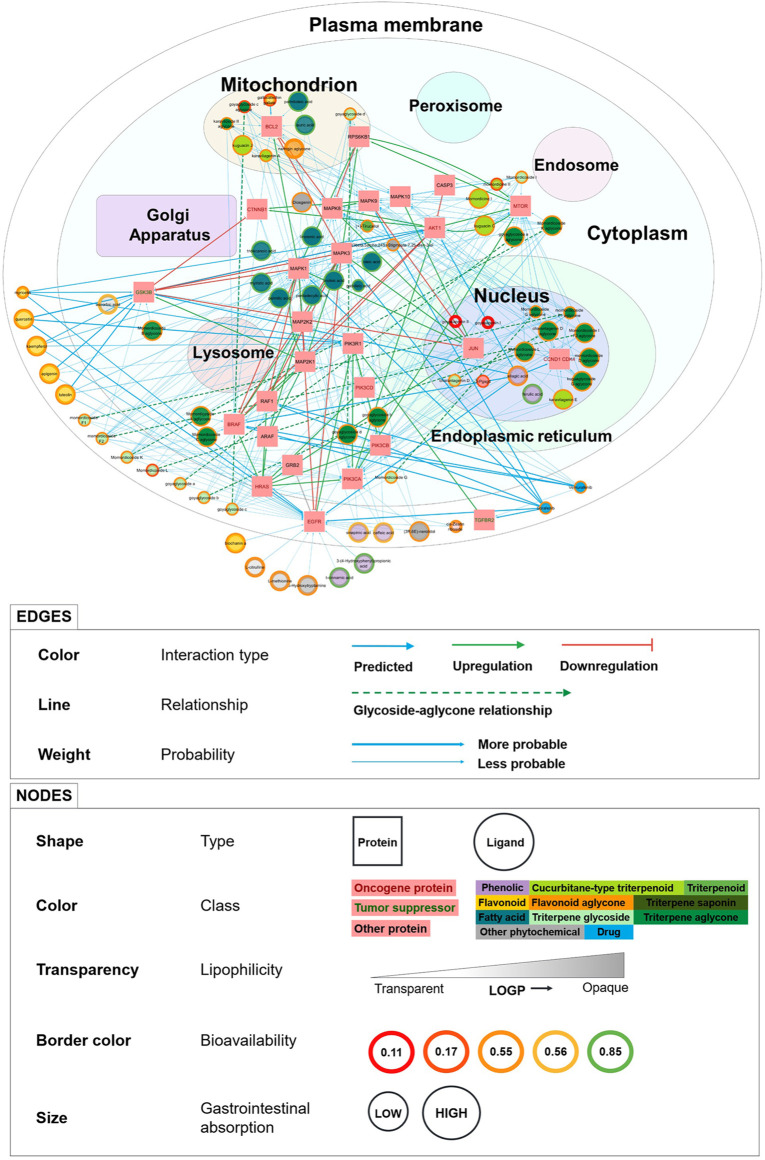
PCPI-SIGNOR disease network visualization for bitter gourd against colorectal cancer. The cell template is from the Cytoscape plug-in boundaryLayout. Detailed legend at the bottom. The network can be interactively viewed at https://yumibriones.github.io/network/.

Supplementary Figure S1 shows the evolution of the network visualization in graphical form. The complete PCPI network and detailed legend are shown in Figure 2 in the Results section. We visualized the network in two ways: with a white background (Figure 2) and a dark background (Supplementary Figure S2).

### 2.6 Evaluation Through Docking

Evaluation of interactions in the “PCPI-SIGNOR disease network visualization” may be done *in silico*, *in vitro*, or *in vivo*. We chose to evaluate PCPIs *in silico* through molecular docking with Autodock Vina, which outperforms its predecessor AutoDock 4 in speed and accuracy (Trott and Olson, 2010).

Protein structures were downloaded from the Protein Data Bank (PDB) (Berman et al., 2000). We used the Auto in silico Consensus Inverse Docking (ACID) server to guide our PDB structure selection (Wang et al., 2019). ACID contains a curated set of protein targets according to the following restrictions:

1) no structures with resolution larger than 3.0 Å;
2) no structures solved by Nuclear Magnetic Resonance (NMR) (structures are all solved by X-ray diffraction for uniformity);
3) no structures with ligands containing nonstandard atoms (e.g. Si, Be); and
4) structures must have only one drug-like ligand bound in the active site.

Structures of the bound inhibitors were obtained from PDB while phytochemical structures were obtained from PubChem. Protein and ligand structures were prepared for docking with Autodock Tools. We manually calculated grid boxes using AutoDock Tools, centering the box on the bound ligand in the active site. In the absence of a bound inhibitor, protein structure was analyzed with Aquaria (http://aquaria.ws/), which aligns UniProt sequence with a chosen PDB structure and highlights features such as binding site (O’Donoghue et al., 2015).

For each protein, we docked the inhibitor bound to the original PDB structure as a positive control before docking phytochemicals. Results were visualized in 3D with ChimeraX (Pettersen et al., 2021) and in 2D with LigPlot+ (Laskowski and Swindells, 2011).

## 3 Results

This section details results for our proof-of-concept study, where we applied the workflow to visualize a PCPI network for bitter gourd against colorectal cancer.

### 3.1 Phytochemical Compilation

We compiled 169 phytochemicals found in the fruit, seeds, and leaves of bitter gourd. These were taken from IMPPAT and reviews by Raina et al. (2016), Jia et al. (2017), and Mozaniel et al. (2018). Most were phenolic acids, triterpene glycosides, and aglycones. For positive controls, we selected the chemotherapy drugs vemurafenib (a selective B-raf inhibitor) and sorafenib (a multi-kinase inhibitor). Meanwhile for negative controls, we chose alprazolam (a benzodiazepine), tolnaftate (an antifungal), and tigecycline (a tetracycline antibiotic), all of which have similar structures to phytochemicals but are not expected to act on colorectal cancer signaling. In total, 174 ligands were compiled for screening. The phytochemical list is shown in Supplementary Table S1 and summarized in Supplementary Figure S3.

### 3.2 Reverse Screening

SwissTargetPrediction predicted 6937 PCPIs with nonzero probability between 166 phytochemicals and 772 protein targets. No matches were found for (+)-catechin, (-)-epicatechin, and the *cis*-zeatin riboside aglycone.

For negative controls, SwissTargetPrediction predicted 52 DPIs for alprazolam, 7 DPIs for tolnaftate, and 17 DPIs for tigecycline with nonzero probability. The top predicted targets for alprazolam were GABA receptors, consistent with experimental knowledge. For tolnaftate and tigecycline, human targets were identified because of structural similarity to other molecules. SwissTargetPrediction may identify false positives, highlighting the need for an evaluation step.

For positive controls, SwissTargetPrediction predicted 100 DPIs for vemurafenib and 100 DPIs for sorafenib, all with nonzero probability. For sorafenib, all results had probability = 1 with targets being mostly protein kinases, consistent with experimental knowledge. For vemurafenib, there were only four results with probability = 1 including the experimentally known target B-Raf proto-oncogene, serine/threonine kinase (B-raf). This demonstrates the reliability of SwissTargetPrediction as a reverse screening tool. The “Complete PCPIs” list is shown in Supplementary Table S2.

### 3.3 Network Building

All g:Profiler results are listed in Supplementary Table S3 with the top ten results shown in Supplementary Figure S4. Pathway enrichment of phytochemical targets identified 23 protein targets in the KEGG colorectal cancer entry. These proteins were involved in the epidermal growth factor receptor (EGFR)/mitogen-activated protein kinase (MAPK) and phosphatidylinositol-4,5-bisphosphate 3-kinase (PI3K)/protein kinase B (Akt) pathways, Wingless-related integration site (Wnt) signaling, apoptosis and cell cycle regulation. The disease-specific protein targets included oncogene proteins like catenin beta 1 (CTNNB1) and B-raf and the tumor suppressor glycogen synthase kinase 3 beta (GSK3b). Protein classifications are listed in Supplementary Table S4.

A separate g:Profiler analysis for the negative controls alprazolam, tolnaftate, and tigecycline found no protein targets involved in the KEGG colorectal cancer pathway. Meanwhile, pathway enrichment of positive controls vemurafenib and sorafenib identified four additional protein targets involved in KEGG colorectal cancer: A-Raf proto-oncogene, serine/threonine kinase (A-Raf), Raf-1 proto-oncogene, serine/threonine kinase (Raf-1), mitogen-activated protein kinase kinase 2 (MAP2K2), and transforming growth factor beta receptor 2 (TGFBR2) (Supplementary Table S3).

In total, the KEGG colorectal cancer PCPI-SIGNOR network contained 98 nodes (69 phytochemicals, 2 drugs, and 27 proteins) and 331 interactions (251 PCPIs, 60 PPIs, 10 DPIs, and 10 glycoside-aglycone relationships). The PCPI network and legend are shown in Figure 2. The dark version of the network can be viewed at https://yumibriones.github.io/network/(Supplementary Figure S2). Supplementary Table S5 contains the data used to build the “Annotated disease-specific PCPI-SIGNOR network.”

### 3.4 Network Visualization

Figure 2 shows the “PCPI-SIGNOR disease network visualization” for bitter gourd against colorectal cancer.

Our PCPI network has a number of advantages over other visualization methods for medicinal plant interactions. The reverse screening tool BATMAN-TCM represents phytochemicals, proteins, pathways and diseases as nodes in a simple network. Yi et al. (2018) have also documented a workflow resulting in a visualization similar to BATMAN-TCM. However, natural products research often aims to study the action of a medicinal plant against a specific disease. These simple visualizations lack the information needed to address this problem, and additional information is presented in other diagrams or in the text of the paper. Meanwhile, our PCPI network presents plenty of information in a single diagram designed to be intuitively understood by biologists.

One clear advantage of our visualization is that nodes are sorted by subcellular compartment, highlighting which phytochemicals have targets in specific organelles (Figure 2). For instance, phytochemicals in the mitochondrion must target B-cell lymphoma 2 (Bcl-2). Seeing subcellular location makes it easier for biologists to identify the roles of proteins in the network.

Another major advantage is the display of pharmacokinetic properties to help assess whether phytochemicals are able to reach protein targets in the cell. High investigation priority may be given to phytochemicals with larger nodes (high GI absorption) and green or orange borders (high or medium bioavailability). Seeing phytochemical classifications is also helpful, as priority can be given to classes such as triterpenoids and flavonoids which are more unique to bitter gourd.

Our visualization also conveys information through edges. The thickest edges (SwissTargetPrediction probability = 1) represent interactions already recorded in ChEMBL. Novel predictions would have thinner edges. We can also see relationships between phytochemicals and their metabolism products by following the dashed arrows. Interactions between proteins are represented with green or red arrows for up or downregulation, revealing the downstream effects of a phytochemical beyond its direct protein target.

To illustrate how these advantages come together, here is an important insight we can get from Figure 2. Triterpene glycosides (light green) are all small nodes mostly in the plasma membrane. However, following the dashed arrows reveals that many aglycone products (dark green) have large nodes and are in the nucleus and cytoplasm. This tells us that aglycones generally have higher GI absorption than glycosides with targets deeper in the cell. This supports experimental knowledge that aglycones are better absorbed than their glycoside parents (Bhattacharya, 2019).

**FIGURE 3:**
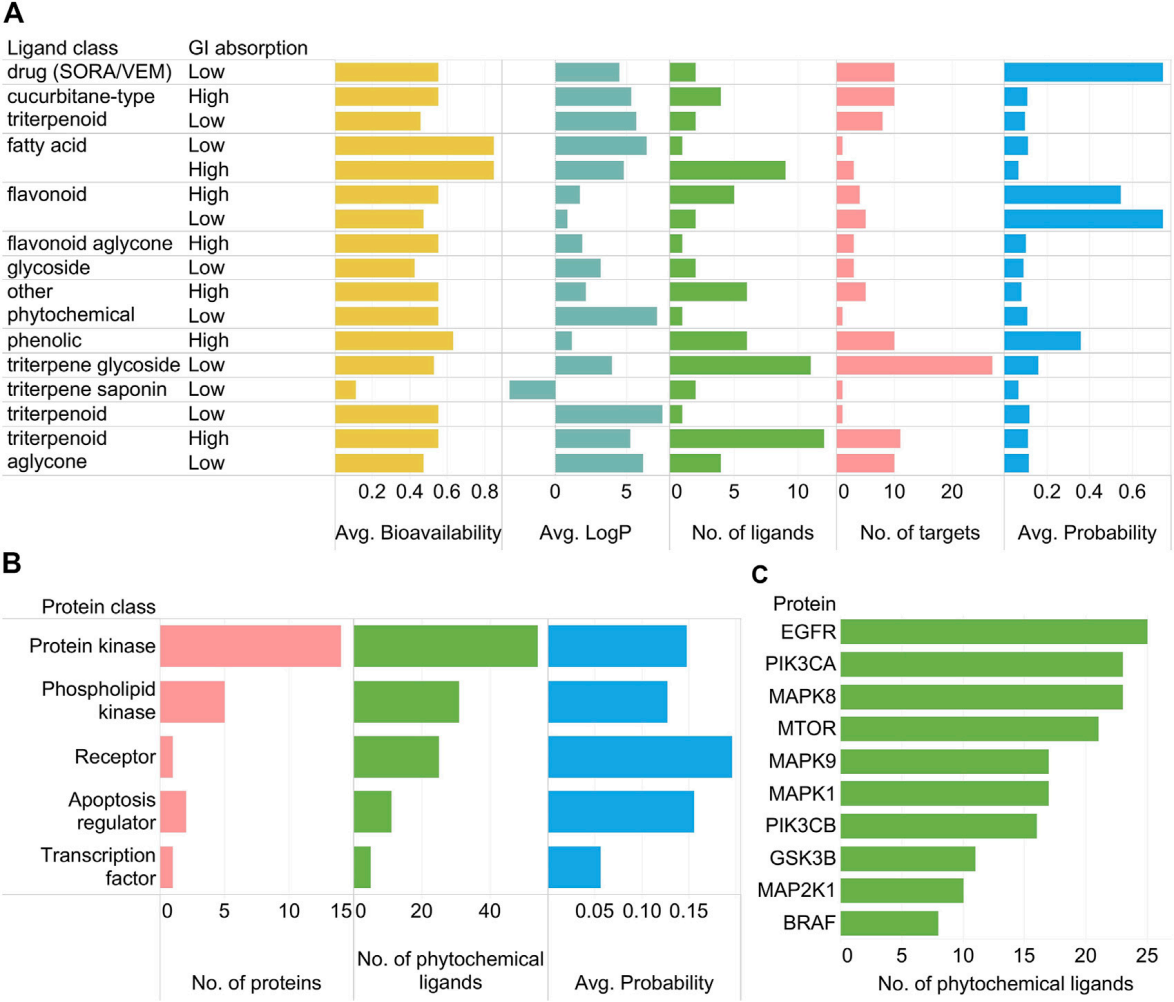
**(A)** Relationships of SwissADME parameters (high/low GI absorption, average bioavailability, average log  *p*) and SwissTargetPrediction results (number of ligands, number of protein targets, average probability) per phytochemical class as well as the two positive control drugs vemurafenib and sorafenib (SORA/VEM) in the KEGG colorectal cancer PCPI-SIGNOR network. **(B)** Number of proteins, number of phytochemical ligands, and average probability scores per protein class. Proteins targeted only by SORA/VEM are not included in the subfigure. **(C)** Top ten most targeted proteins by phytochemical ligands (excluding SORA/VEM).

Important trends in the PCPI network can be summarized using standard bar graphs as in Figure 3.

As observed in Figure 2, triterpene glycosides were highly abundant but had low GI absorption while aglycones had high GI absorption (Figure 3A). Protein kinases were abundant and highly targeted by phytochemicals (Figure 3B). Highly targeted proteins include EGFR and the mechanistic target of rapamycin kinase (mTOR) (Figure 3C), though this is already apparent from Figure 2. While bar graphs can reveal general trends in the data, the network visualization shows these trends while also showing specific interactions. Figure 2 alone can already highlight PCPIs to evaluate further *in vitro*, *in vivo*, or *in silico*.

### 3.5 Evaluation by Molecular Docking

We used Autodock Vina (Vina hereafter) to dock 28 PCPIs and 6 DPIs in the KEGG colorectal cancer PCPI-SIGNOR network. We chose phytochemicals with high GI absorption from various classes including phenolic acids, triterpenoids, flavonoids, fatty acids, and aglycones. Proteins were selected from the EGFR/MAPK and PI3K/Akt pathways, Wnt signaling, apoptosis, and the cell cycle. Only the top pose from Vina was considered. Detailed docking information is listed in Supplementary Table S6.

For positive docking controls, we docked each protein to its bound inhibitor from the PDB structure. We found that predicted poses from Vina were visually similar to experimental poses. Docking interaction energies were generally more negative for bound inhibitor-protein pairs versus phytochemical-protein pairs (Supplementary Figure S5). The positive controls vemurafenib and sorafenib docked with highly negative energies comparable to the bound inhibitors. We concluded that Vina predicted binding poses with fairly high accuracy.

Flavonoids and phenolics docked to the adenosine triphosphate (ATP)-binding sites of protein kinases with highly negative docking interaction energies, suggesting competitive inhibition of kinase activity. On the other hand, triterpenoids generally had less negative docking interaction energies when docked to the ATP-binding site. This suggests that flavonoids and phenolics have a high potential for *in vitro* or *in vivo* activity.

To quantify this, we assigned confidence levels to PCPIs based on docking interaction energy (“docking confidence” hereafter) (Supplementary Figure S6). Among the PCPIs with probability = 1, we set the most negative docking interaction energy as the “soft cutoff” (−7.8 kcal/mol). The upper bound of the 99.7% confidence interval (CI) (−6.4 kcal/mol) was set as the “hard cutoff.” Interactions were classified as follows:

1) High docking confidence: *docking interaction energy*, *E* < − 7.8 *kcal*/*mol* (soft cutoff);
2) Medium docking confidence: − 7.8 < *E* < − 6.4 *kcal*/*mol* (hard cutoff);
3) Low docking confidence: *E* > − 6.4 *kcal*/*mol*.

Most flavonoid-protein interactions had high docking confidence while triterpenoid-protein interactions had low docking confidence (Supplementary Figure S7). Interestingly however, all interactions between triterpenoids and mTOR had high docking confidence.

We then used docking confidence to calculate “probability confidence” regions based on SwissTargetPrediction probability. We took the mean probability values of each docking confidence level and calculated the 68% CI (equivalent to 1 standard deviation) for each mean (Supplementary Figure S8). Detailed calculations are shown in Supplementary Table S6. Probability confidence regions were assigned as follows:

1) High probability confidence: *probability*, *P* > 0.1263 (upper bound of the mean probability of low docking confidence interactions);
2) Uncertain probability confidence: 0.1263 > *P* > 0.0774 (lower bound of the mean probability of medium docking confidence interactions);
3) Low probability confidence: *P* < 0.0774.

We then sorted each interaction in the KEGG colorectal cancer PCPI-SIGNOR network according to probability confidence regions (Supplementary Table S5). Flavonoids were most abundant in the high probability confidence region, triterpenoid aglycones were abundant in the uncertain region, and triterpene glycosides were abundant in the low probability confidence region. Protein kinases were highly targeted in all probability confidence regions (Supplementary Figure S9).

Docking results are color-coded according to the legend in Supplementary Figure 10, and all visualizations are shown in Supplementary Figures S11–27.

### 3.6 Simplified PCPI Network for Anticancer Action of Bitter Gourd

**FIGURE 4:**
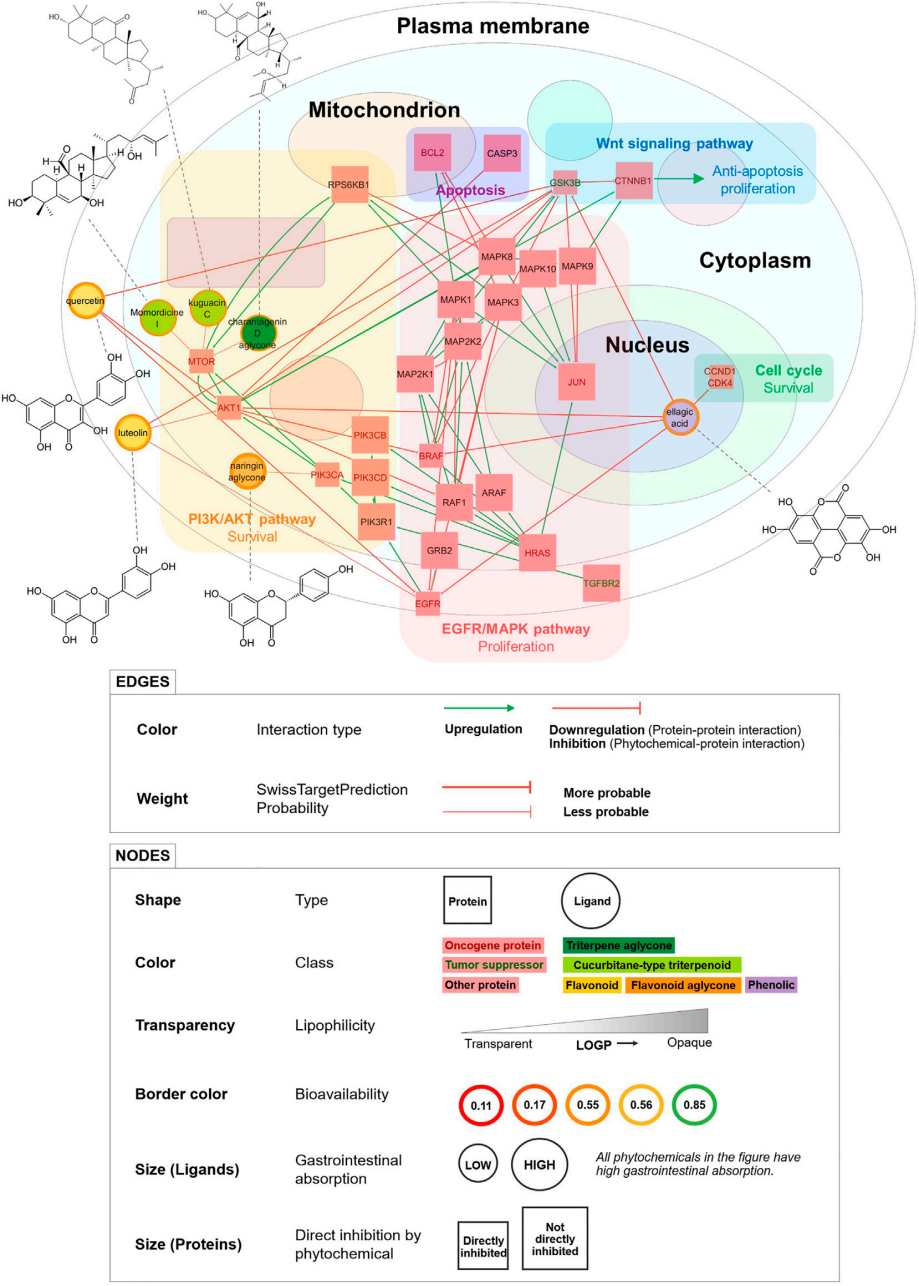
Simplified PCPI network showing the predicted anticancer action of bitter gourd (with high docking confidence and probability = 1 PCPIs from docking). Pathways and downstream effects are shown. Phytochemical structures are connected to their corresponding nodes by dashed lines. All PCPIs are represented by red arrows indicating inhibition. Smaller square nodes are proteins directly inhibited by phytochemicals in the network.

We visualized a smaller PCPI network including only high docking confidence and probability = 1 interactions (Figure 4). This is a simplified model of the predicted anticancer action of bitter gourd. Phytochemicals in this diagram are strong candidates for *in vitro* and *in vivo* activity.

Ellagic acid, a phenolic compound, was predicted to inhibit the most proteins and pathways including the cell cycle, EGFR/MAPK pathway, and PI3K/Akt pathway. Ellagic acid was also predicted to inhibit the tumor suppressor GSK3b, but interestingly, experiments show that inhibition of GSK3b may in fact decrease cancer cell proliferation (Marchand et al., 2012). Meanwhile, the flavonoids quercetin and luteolin were predicted to inhibit the same proteins and pathways including PI3K/Akt and EGFR/MAPK, thereby inhibiting cell survival and proliferation. The triterpenoids momordicine I, kuguacin C, and the charantagenin D aglycone were all predicted to inhibit the PI3K/Akt pathway via mTOR. We highly recommend that these predicted interactions be studied further through *in vitro* and *in vivo* experiments. The phytochemicals in Figure 4 may also be used as marker compounds for medicinal formulations of bitter gourd.

This figure demonstrates the ability of our workflow to visualize high-confidence PCPI predictions as a detailed yet intuitive network. The workflow can be used to create PCPI networks for other medicinal plants and diseases. If small molecule drugs are searched together with medicinal plants, the PCPI network can even identify shared protein targets and potential interaction effects. Unlike the integrated tool BATMAN-TCM, our modular workflow allows researchers to use other tools at any step. However, we recommend using the tools presented in this study as these were carefully selected. The workflow and links to all resources are available at https://yumibriones.github.io/network/workflow.html.

## 4 Conclusion

We developed a novel workflow to visualize the predicted cellular action of a medicinal plant against a disease. We combined select tools into a five-step pipeline: phytochemical compilation, reverse screening, network building, network visualization, and evaluation. The resulting phytochemical-protein interaction (PCPI) network visually reflects protein-protein interactions, subcellular location, phytochemical class, pharmacokinetic data, and other attributes in a single figure. By clearly communicating all these attributes visually, the network helps users identify interactions worth evaluating further. Our proof-of-concept study on bitter gourd against colorectal cancer identified triterpenoid aglycones and flavonoids as key players in the network. The PCPI network and workflow are available at https://yumibriones.github.io/network/. We evaluated select PCPIs through docking to produce a smaller network of high-confidence interactions that can be validated *in vitro* and *in vivo*. Overall, this workflow streamlines natural products research by using readily available tools to visualize a rich, intuitive PCPI network.

## Data Availability

The original contributions presented in the study are included in the article/Supplementary Material, further inquiries can be directed to the corresponding authors.
