# Supplementary material for: Visualizing Phytochemical-Protein Interaction Networks: Momordica charantia and Cancer
Source: Front Bioinform. 2021 Dec 13;1:768886. doi: 10.3389/fbinf.2021.768886 (PMC9580883; doi:10.3389/fbinf.2021.768886)
Supplement: Supplementary file 1 [file DataSheet1.PDF]

Supplementary Material

1 SUPPLEMENTARY TABLES

Supplementary Tables S1, S2, S3, S4, S5, and S6 are included in a separate Excel file entitled "Supplementary_Tables.xlsx".

Table S1. Phytochemical list. Includes 174 ligands (169 phytochemicals, 2 positive control drugs, 3 negative control drugs) in bitter gourd. The "Reference" column contains "IMPPAT" if the phytochemical was taken from the Indian Medicinal Plants, Phytochemistry And Therapeutics database. For phytochemicals taken from literature reviews by Jia et al. (2017), Mozaniel et al. (2018) or Raina et al. (2016), the reference column contains "jia", "mozaniel", or "raina" respectively.

Table S2. Complete PCPIs. Includes 7137 SwissTargetPrediction results (6937 from phytochemicals, 200 from positive controls, 76 from negative controls) with probability scores greater than zero for 166 phytochemicals, 2 positive controls, and 3 negative controls. No results were predicted for three phytochemicals, namely (+)-catechin, (-)-epicatechin, and the cis-zeatin riboside aglycone. Each row represents a unique PCPI. The "Probability" column indicates the calculated probability of the interaction. The "Known Actives (3D/2D)" column indicates the number of ligands in the ChEMBL database considered to match the phytochemical based on 3D and 2D similarity.

Table S3. Results for pathway enrichment using g:Profiler using KEGG as the data source. Enrichment was performed for the predicted protein targets of phytochemicals, positive controls, and negative controls. The negative logarithm of the adjusted p-value evaluates the statistical enrichment of the pathway in the set of proteins. Rows containing "Colorectal cancer" (rows 73 and 222) are highlighted in yellow. Intersected proteins in these rows are used to filter the complete PCPIs to disease-specific PCPIs and protein targets.

Table S4. Disease-specific protein targets. List of the 27 protein targets (23 targeted by phytochemicals, 4 targeted by positive control drugs only) involved in colorectal cancer based on g:Profiler analysis. For each protein, we indicated the full name, protein class, cellular location, cancer function, and whether it is targeted by a phytochemical or drug.

Table S5. Annotated PCPI-SIGNOR disease network. Contains all information needed to build the KEGG colorectal cancer PCPI-SIGNOR network for bitter gourd in colorectal cancer. The network contains 251 phytochemical-protein interactions (PCPIs), 60 protein-protein interactions (PPIs), and 10 drug-protein interactions (DPIs). All columns ending

in “A” or “B” are attributes of “Entity A” and “Entity B” respectively. Columns ending in “A” include information on class, SMILES, and pharmacokinetic data. Meanwhile, columns ending in “B” include information on protein class and location. The column “Effect” indicates the type of interaction: either “predicted” (from SwissTargetPrediction), “up-regulates” or “down-regulates” (from SIGNOR 2.0). All succeeding columns are edge attributes such as SwissTargetPrediction probability, SIGNOR mechanism and PPI scores, and docking interaction energies for selected interactions. Rows are colored based on the probability confidence of their interaction, for which calculations are shown in Table S6 (blue: probability = 1, green: high probability confidence, gray: uncertain, red: low probability confidence, and white: PPI).

Table S6. Evaluation results. Includes AutoDock Vina output for 43 ligand-protein pairs. Docking interaction energy and calculated K_d are shown for each pair. A white background color means the interaction in the figure is between a protein and its bound inhibitor (positive docking control). A dark blue background indicates a drug-protein interaction (DPI) with probability = 1. Meanwhile, a light blue background indicates a phytochemical-protein interaction (PCPI) with probability = 1. These probability = 1 interactions were used to calculate the soft and hard cutoffs for docking interaction energies. A green background indicates a high docking confidence interaction, or $docking\ interaction\ energy,\ E < -7.8\ kcal/mol$ (below soft cutoff). An orange background indicates a medium docking confidence interaction, or $-7.8\ kcal/mol < E < -6.4\ kcal/mol$ (between soft and hard cutoffs). A red background indicates a low docking confidence interaction, or $E > -6.4\ kcal/mol$ (above hard cutoff). The Excel sheet also contains calculations for hard and soft cutoffs, as well as calculations for probability confidence levels based on SwissTargetPrediction probability scores (used to assign probability confidence levels in Table S5).

2 SUPPLEMENTARY FIGURES

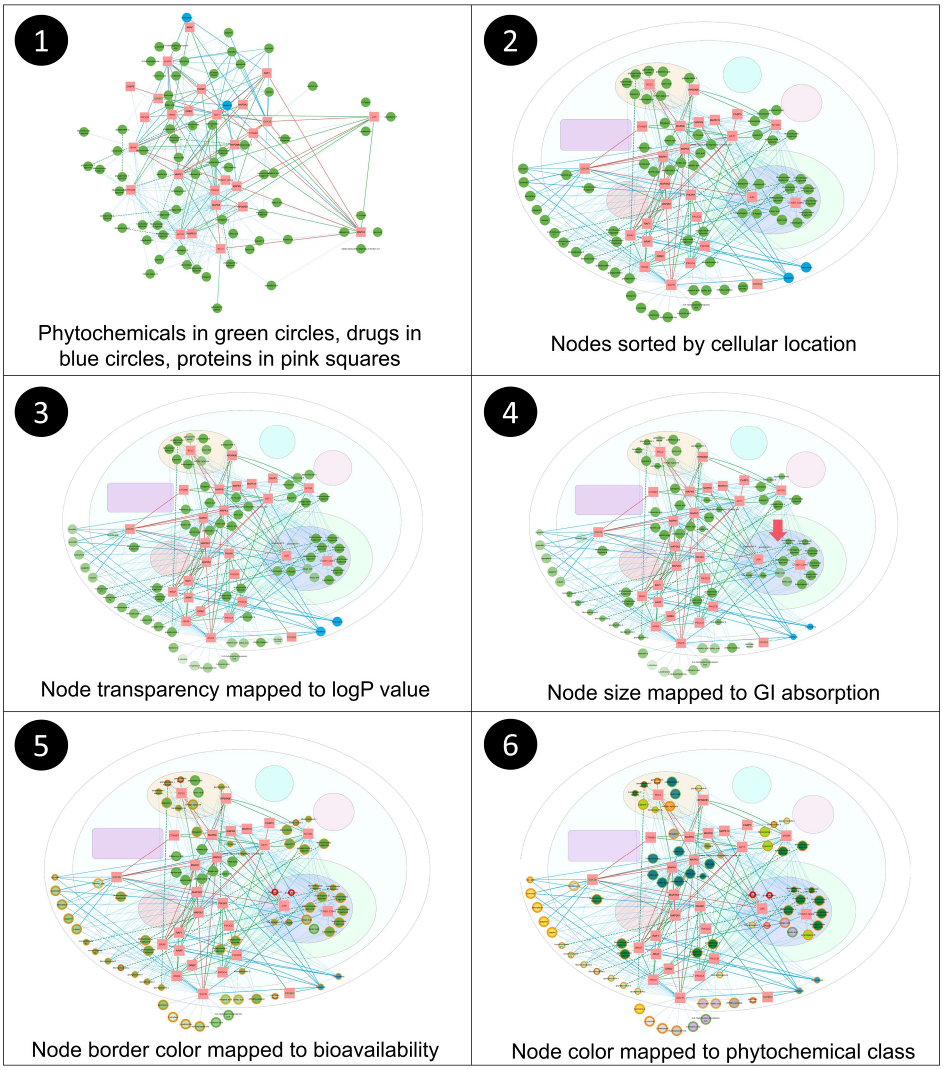

Figure S1. Evolution of the PCPI-SIGNOR disease network visualization. Nodes were initially unorganized with phytochemicals in green, drugs in blue, and proteins in pink. Using boundaryLayout, nodes were sorted by subcellular location. Node transparency was mapped to $\log P$ value, with higher $\log P$ being more opaque. Node size was mapped to gastrointestinal (GI) absorption, with higher absorption being larger. Node border color was mapped to bioavailability scores, with red for the lowest and green for the highest scores. Finally, phytochemical nodes were colored based on their classification.

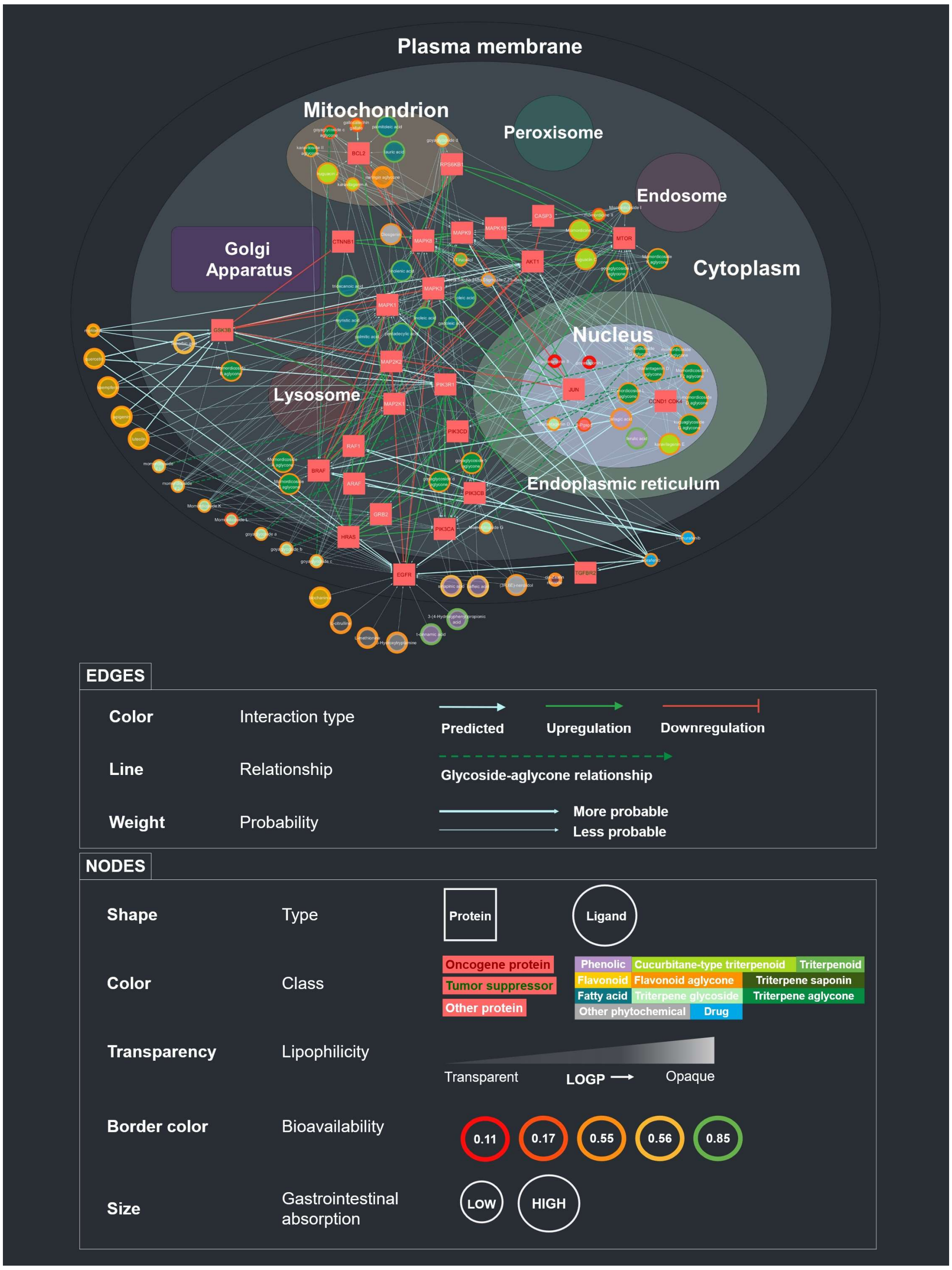

Figure S2. Dark version of the PCPI-SIGNOR network for bitter gourd in colorectal cancer, visualized with Cytoscape. The network is organized within a cell template from the boundaryLayout plug-in. Detailed legend is at the bottom of the figure. Available for interactive viewing at `https://yumibriones.github.io/network/`.

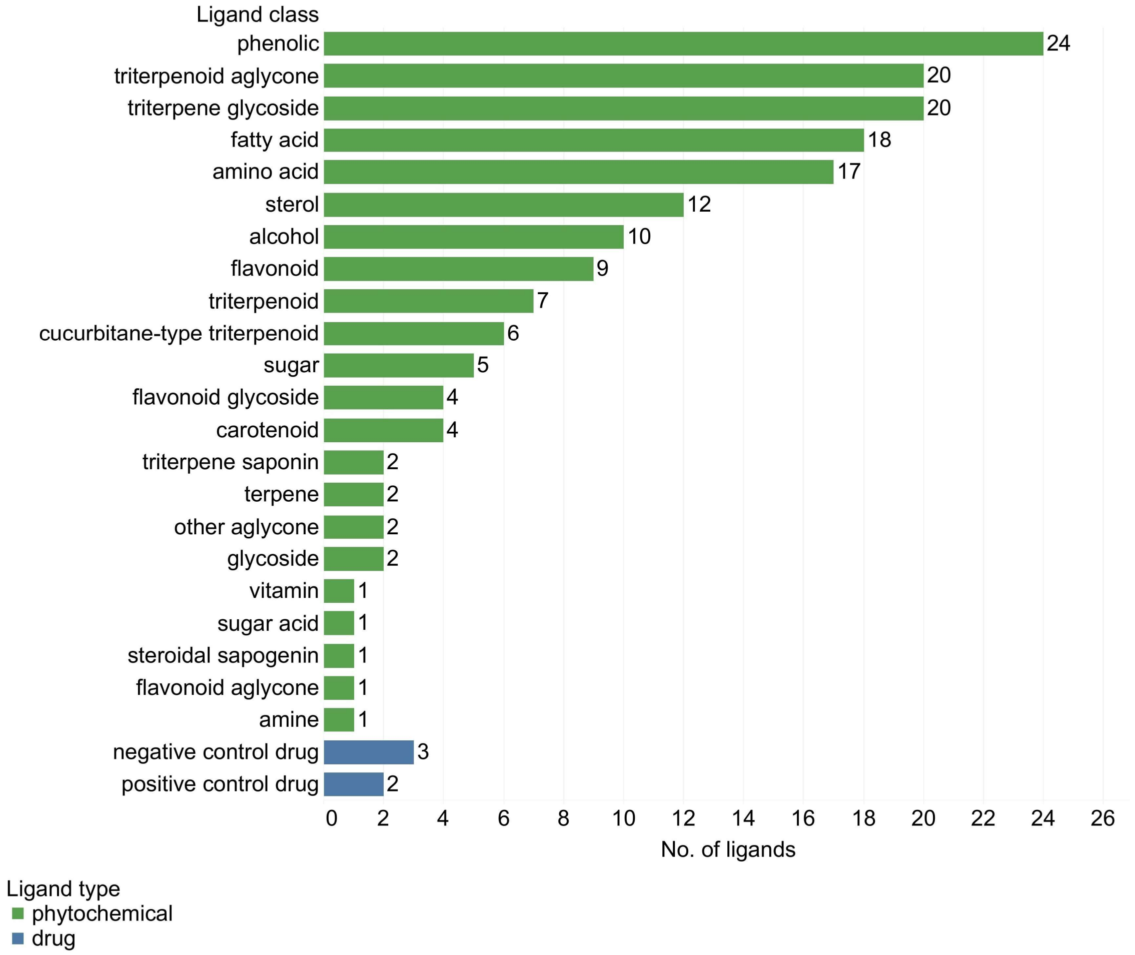

Figure S3. Graph showing the distribution of the 174 phytochemicals in bitter gourd among various phytochemical classes, including the 2 positive control drugs sorafenib and vemurafenib (shortened to SORA/VEM in the figure).

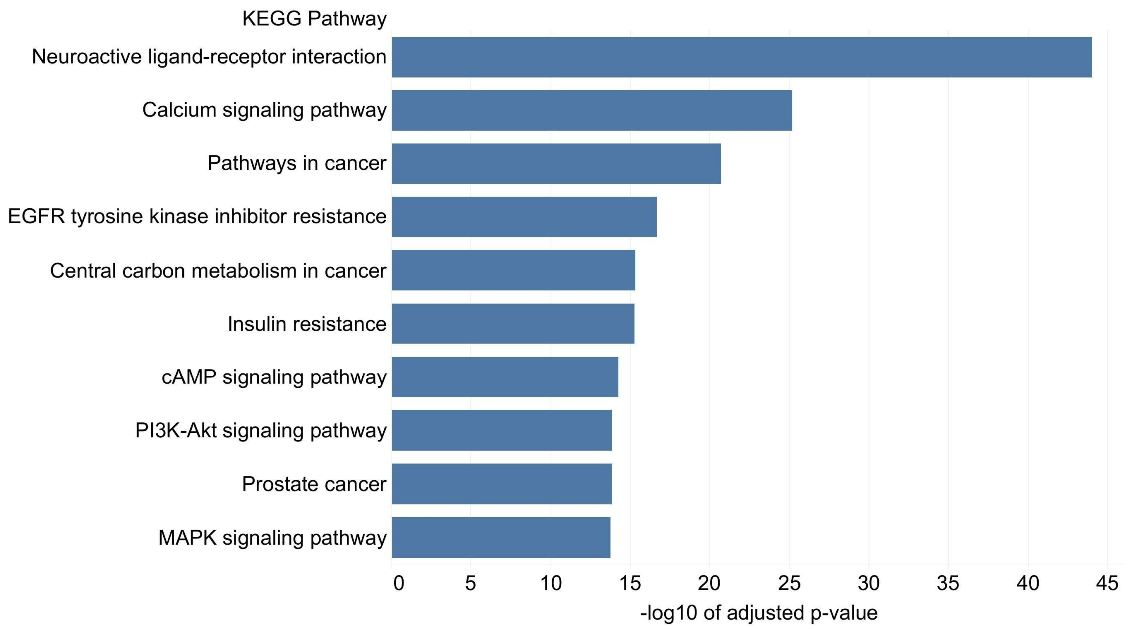

Figure S4. Top ten statistically enriched pathways in the set of 772 protein targets of phytochemicals predicted by SwissTargetPrediction. Enrichment analysis was performed with g:Profiler using KEGG as the reference database.

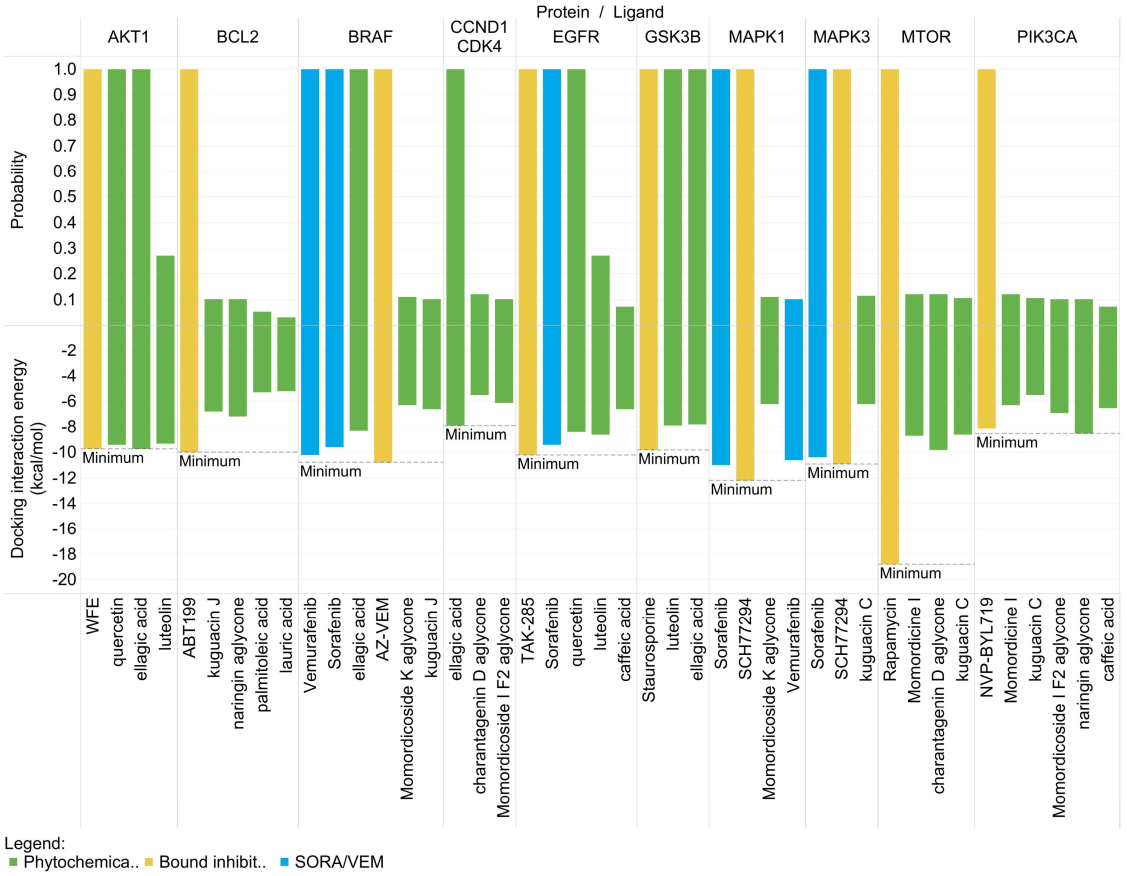

Figure S5. Protein-focused bar chart showing relationship of SwissTargetPrediction probability (top half of graph) and docking interaction energy from Autodock Vina (bottom half of graph) for the 43 chosen ligand-protein pairs. Yellow bars are bound inhibitors of the protein taken from the chosen PDB structure, blue bars are sorafenib or vemurafenib (SORA/VEM), and green bars are phytochemicals. Probability scores were taken from SwissTargetPrediction except for bound inhibitors which were automatically assigned a probability of 1. Dashed lines are drawn at the minimum (most negative) value of docking interaction energy for each protein.

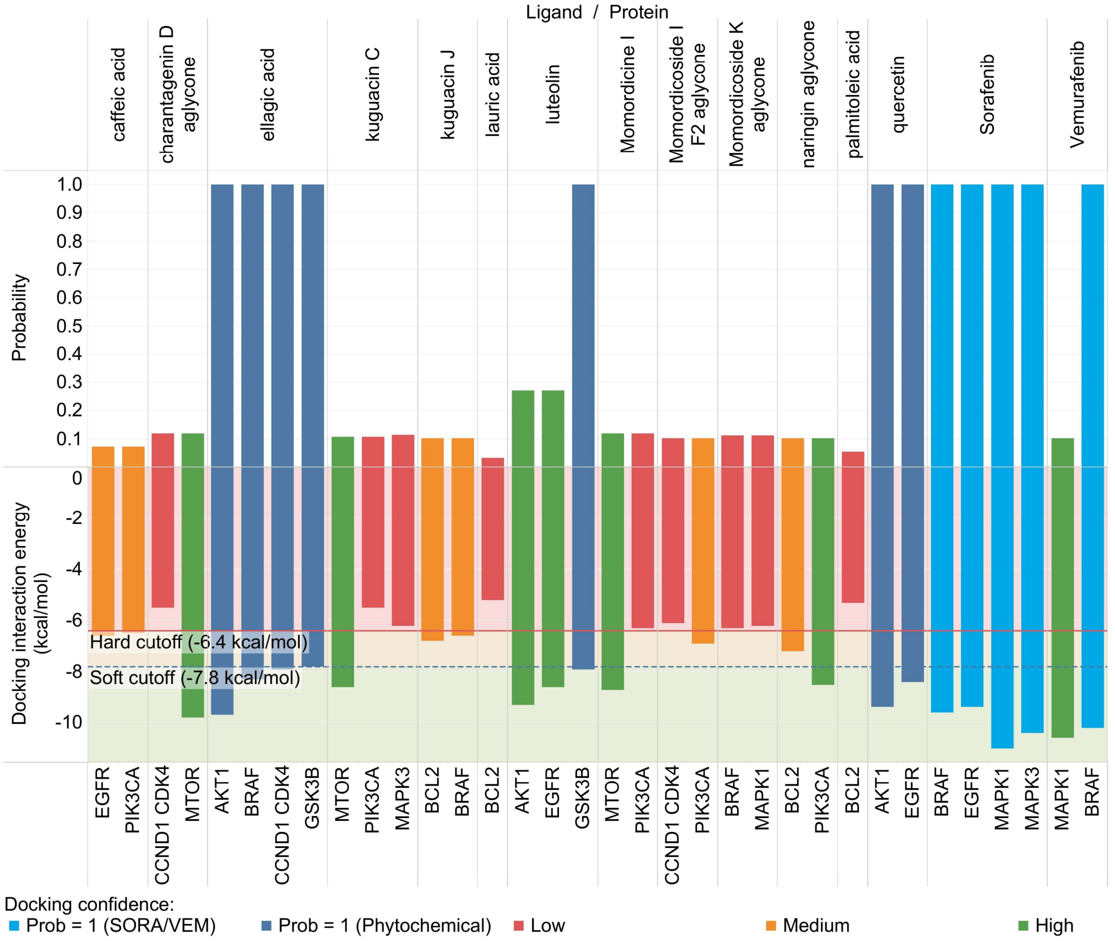

Figure S6. Phytochemical-focused bar chart showing relationship of SwissTargetPrediction probability (top half of graph) and docking interaction energy from Autodock Vina (bottom half of graph) for phytochemical-protein and drug-protein pairs. The dashed blue line represents the "soft cutoff" at -7.8 kcal/mol. The solid red line represents the "hard cutoff" at -6.4 kcal/mol. These cutoffs were calculated as shown in Table S6 from the phytochemicals with probability = 1 (dark blue bars). Bright blue bars represent sorafenib/vemurafenib (SORA/VEM) with probability = 1. Green bars represent high docking confidence interactions with $docking\ interaction\ energy,\ E < -7.8\ kcal/mol$ (below soft cutoff). Orange bars represent medium docking confidence interactions with $-7.8\ kcal/mol < E < -6.4\ kcal/mol$ (between soft and hard cutoffs). Red bars represent low docking confidence interactions with $E > -6.4\ kcal/mol$ (above hard cutoff).

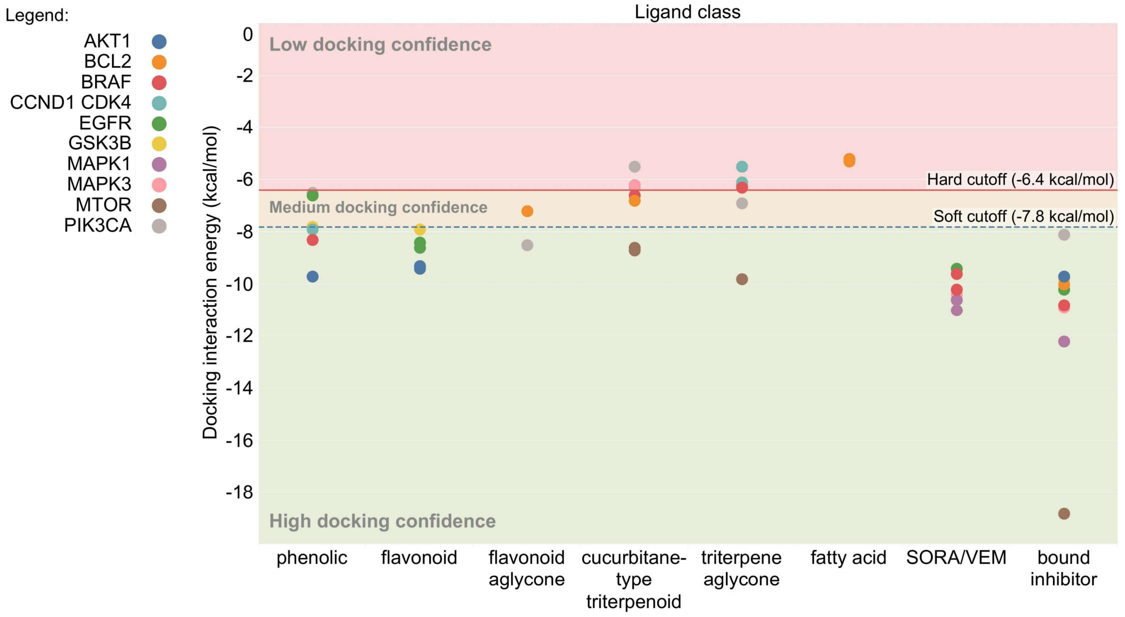

Figure S7. Dot plot showing docking interaction energies between proteins and phytochemical classes or sorafenib/vemurafenib (SORA/VEM). Classifications of phytochemicals selected for docking are indicated in Table S6. Dots are colored based on the protein being docked to (legend on the left). The light green region bounded by the soft cutoff (dashed blue line at -7.8 kcal/mol) contains all high docking confidence interactions. The light orange region between the soft and hard cutoff lines contain all medium doking confidence interactions. The light red region bounded by the hard cutoff (solid red line at -6.4 kcal/mol) contains all low docking confidence interactions.

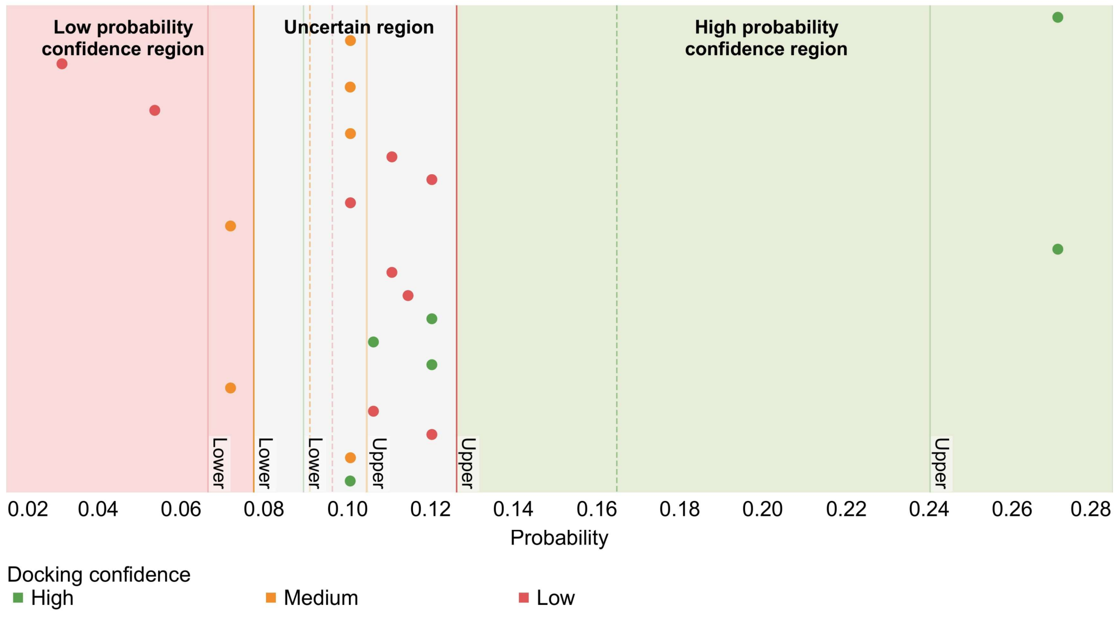

Figure S8. Dot plot showing probability confidence regions calculated from SwissTarget-Probability scores. Each dot represents an individual PCPI investigated through docking, excluding those with probability = 1. Dots are colored based on their docking confidence level (green = high, orange = medium, red = low). The x-axis contains SwissTargetPrediction probability values. Dashed lines are drawn at the mean probability value of each docking confidence level (i.e. green dashed line = mean probability of high docking confidence interactions, and so on). Solid lines are drawn at the upper and lower bounds of the 68% confidence interval (CI) (i.e. green solid lines = upper and lower bounds of the 68% CI for the mean probability of high docking confidence interactions, and so on). The upper bound of low docking confidence interactions (probability = 0.1263) is the boundary for the high probability confidence region. All interactions with $probability,\ P > 0.1263$ are of high probability confidence. Below the boundary, in the gray region ($0.1263 > P > 0.0774$), there is a mix of high, medium, and low docking confidence interactions. This is thus termed the uncertain region. The lower bound of medium confidence interactions (probability = 0.0774) is the boundary between the uncertain and low probability confidence regions. Interactions with $P < 0.0774$ have low probability confidence. Detailed calculations for the probability confidence regions are shown in Table S6.

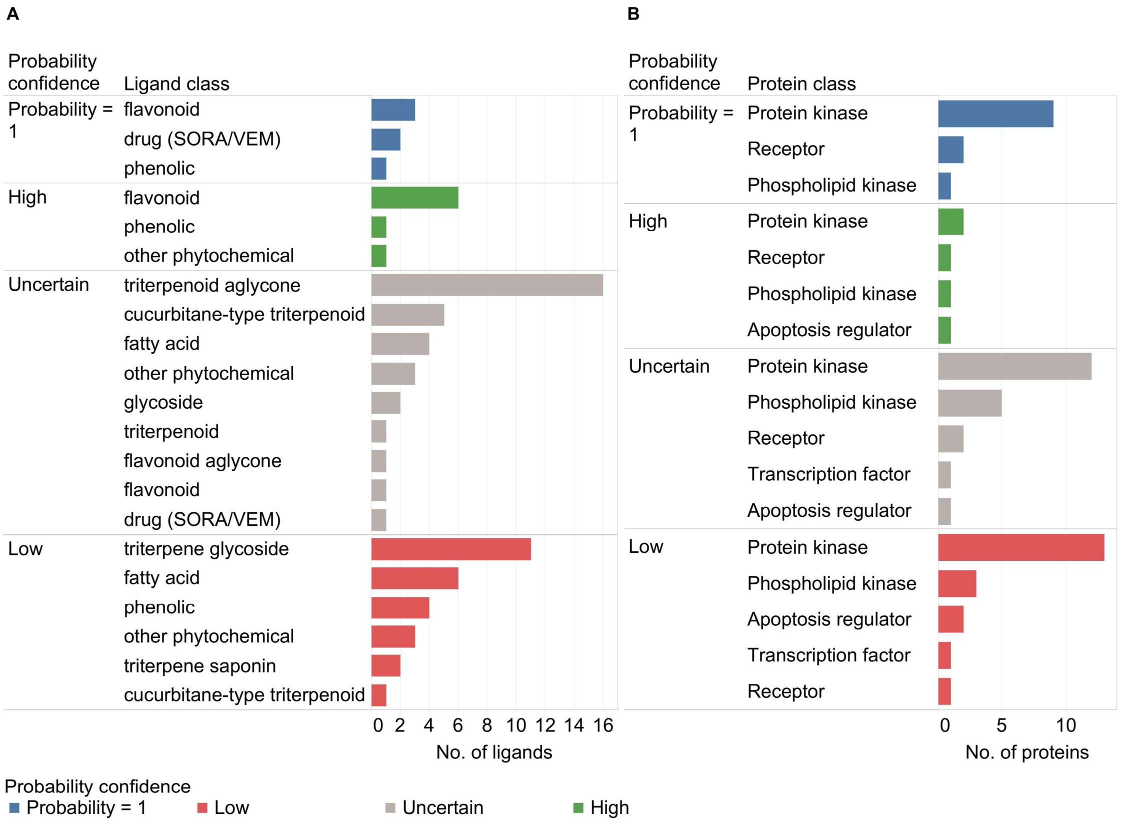

Figure S9. Bar chart showing probability confidence levels of phytochemical and protein classes in the KEGG colorectal cancer PCPI-SIGNOR network. Classifications are shown in Table S5. **(A)** Bar chart showing phytochemical classes per probability confidence region. Flavonoids are most abundant in the high probability confidence region. Aglycones are most abundant in the uncertain region. Triterpene glycoside are most abundant in the low probability confidence region. **(B)** Bar chart showing protein classes per probability confidence region. Protein kinases were most abundant in all probability confidence regions.

Background colors for succeeding figures:

For docking interaction energy, *E*

Phytochemicals:

Phytochemical with Probability = 1
Used to calculate soft (-7.8 kcal/mol) and hard (-6.4 kcal/mol) cutoffs

High docking confidence
E < -7.8 kcal/mol

Medium docking confidence
-7.8 kcal/mol < *E* < -6.4 kcal/mol

Low docking confidence
E > -6.4 kcal/mol

Bound inhibitors and SORA/VEM:

Positive docking control
Bound inhibitor from PDB structure

SORA/VEM with Probability = 1
Vemurafenib or sorafenib with probability = 1

SORA/VEM with High docking confidence
Vemurafenib or sorafenib with *E* < -7.8 kcal/mol

Figure S10. Legend explaining the background colors for docking figures which contain visualizations of docked ligand-protein pairs. For phytochemicals, a light blue background means the interaction in the figure is between a phytochemical and protein with SwissTargetPrediction probability = 1. These probability = 1 interactions were used to calculate the soft and hard cutoffs for docking interaction energies. A light green background means the interaction in the figure is of high docking confidence, or $docking\ interaction\ energy,\ E < -7.8\ kcal/mol$ (below soft cutoff). An orange background means the interaction in the figure is of medium docking confidence, or $-7.8\ kcal/mol < E < -6.4\ kcal/mol$ (between soft and hard cutoffs). A red background means the interaction in the figure is of low docking confidence, or $E > -6.4\ kcal/mol$ (above hard cutoff). For ligands other than phytochemicals, a white background color means the interaction in the figure is between a protein and its bound inhibitor (positive docking control). A dark blue background indicates a probability = 1 interaction between sorafenib or vemurafenib (SORA/VEM) and a protein. Finally, a dark green background indicates a SORA/VEM-protein interaction classified as high docking confidence according to the cutoff scores above.

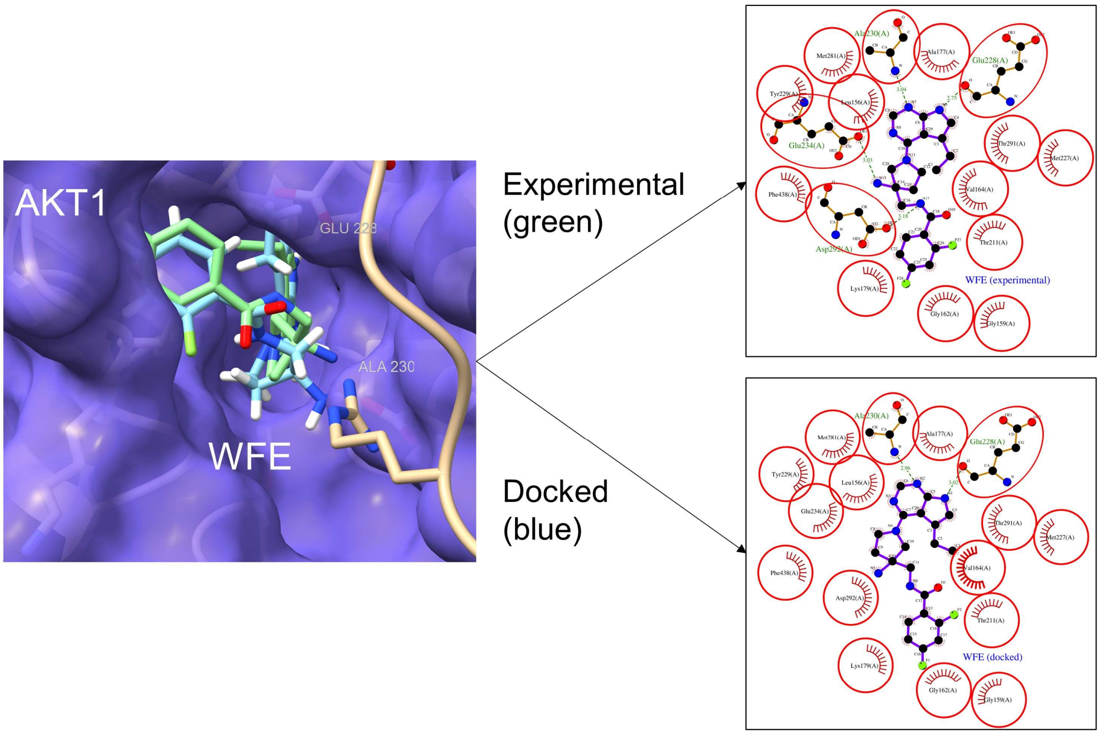

Figure S11. Comparison of experimental and docked pose of bound inhibitor WFE to AKT1 (PDB code: 3MVH) as a positive docking control. Left: ChimeraX visualization of experimental pose (in green) overlaid with docked pose (in blue). Top right: LigPlot+ visualization of experimental pose. Bottom right: LigPlot+ visualization of docked pose. For ChimeraX visualizations, dashed blue lines represent hydrogen bonds. For LigPlot+ visualizations, dashed green lines represent hydrogen bonds, half moons indicate van der Waals interactions, and red circles indicate that the residue has common interactions with other ligands in the figure.

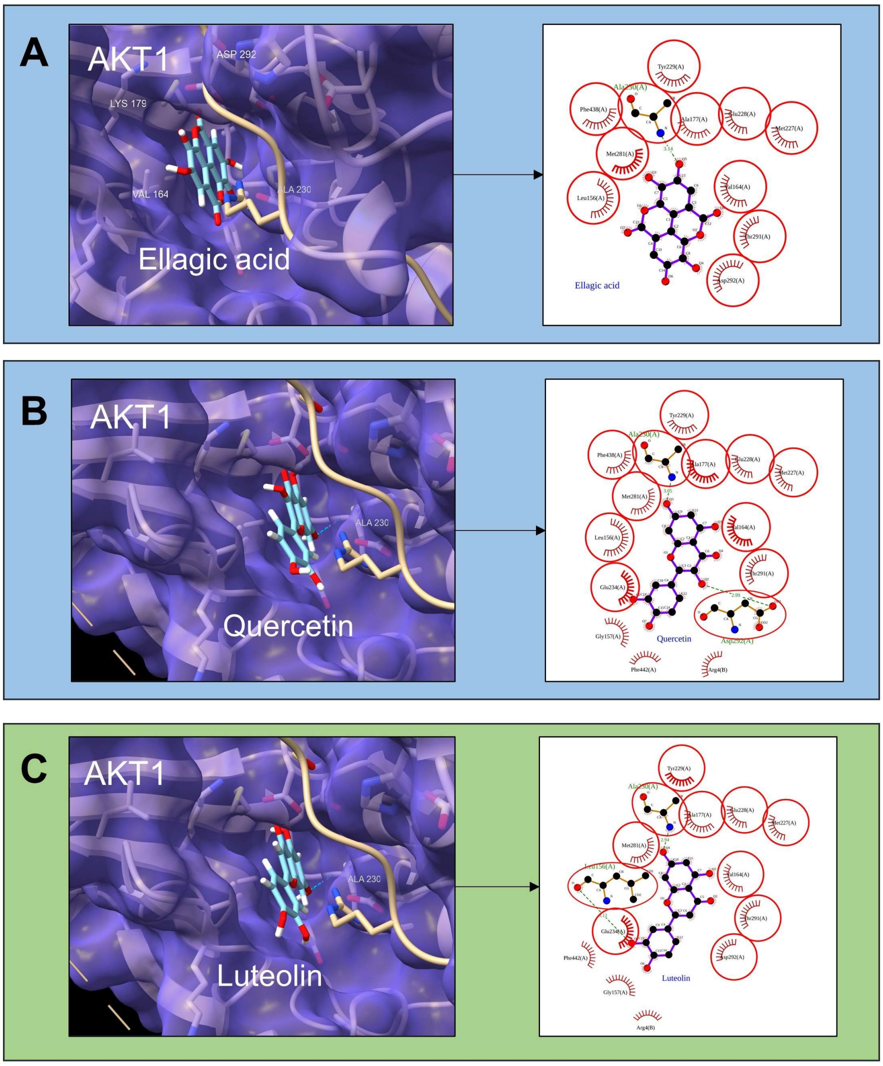

Figure S12. Visualizations of the three phytochemicals docked to AKT1 (PDB code: 3MVH). Subfigure backgrounds are colored based on the docking confidence level of the interaction (see Figure S10 for detailed legend). Left panels: ChimeraX visualizations. Right panels: LigPlot+ visualizations. The phytochemical-protein pairs in each subfigure are as follows: **(A)** ellagic acid and AKT1 (probability = 1), **(B)** quercetin and AKT1 (probability = 1), and **(C)** luteolin and AKT1 (high docking confidence). For ChimeraX visualizations, dashed blue lines represent hydrogen bonds. For LigPlot+ visualizations, dashed green lines represent hydrogen bonds, half moons indicate van der Waals interactions, and red circles indicate that the residue has common interactions with other ligands in the figure.

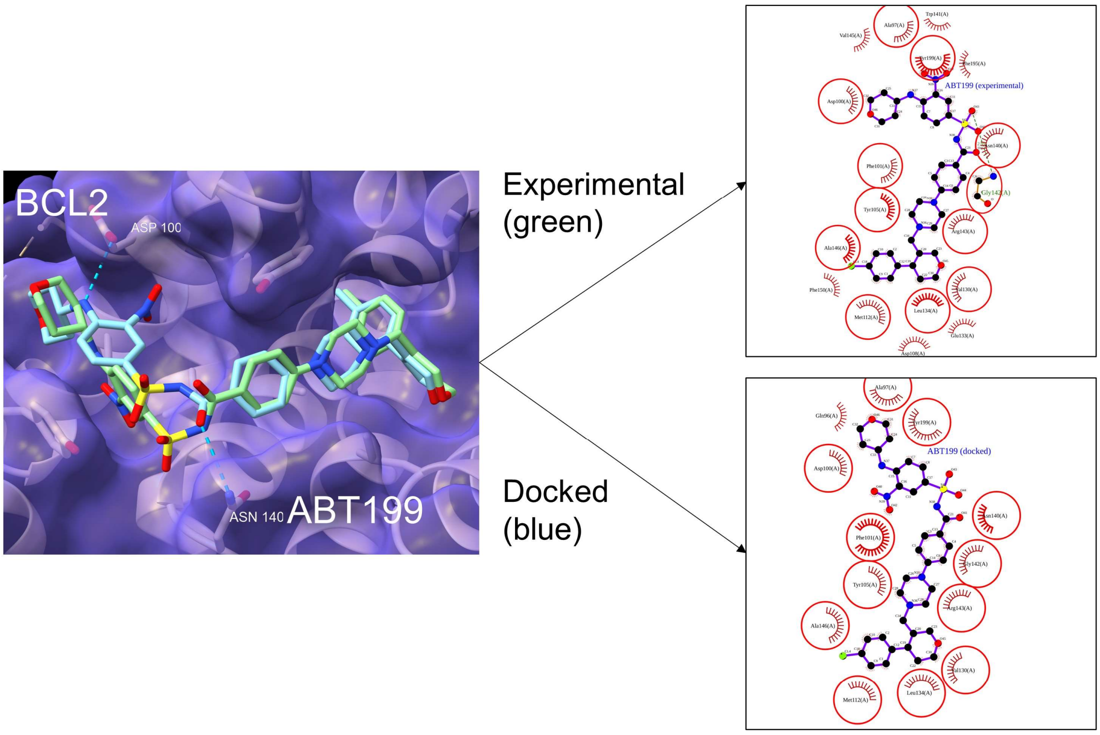

Figure S13. Comparison of experimental and docked pose of bound inhibitor ABT199 to BCL2 (PDB code: 4LXD) as a positive docking control. Left: ChimeraX visualization of experimental pose (in green) overlaid with docked pose (in blue). Top right: LigPlot+ visualization of experimental pose. Bottom right: LigPlot+ visualization of docked pose. For ChimeraX visualizations, dashed blue lines represent hydrogen bonds. For LigPlot+ visualizations, dashed green lines represent hydrogen bonds, half moons indicate van der Waals interactions, and red circles indicate that the residue has common interactions with other ligands in the figure.

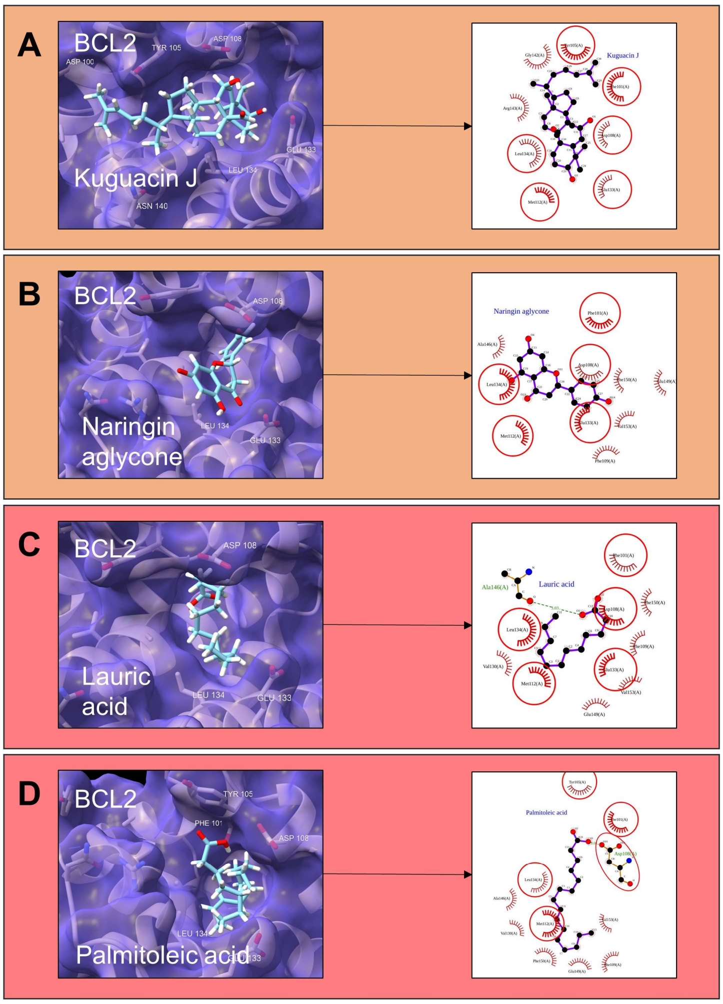

Figure S14. Visualizations of the four phytochemicals docked to BCL2 (PDB code: 4LXD). Subfigure backgrounds are colored based on the docking confidence level of the interaction (see Figure S10 for detailed legend). Left panels: ChimeraX visualizations. Right panels: LigPlot+ visualizations. The phytochemical-protein pairs in each subfigure are as follows: **(A)** kuguacin J and BCL2 (medium docking confidence), **(B)** naringin aglycone and BCL2 (medium docking confidence), **(C)** lauric acid and BCL2 (low docking confidence), and **(D)** palmitoleic acid and BCL2 (low docking confidence). For ChimeraX visualizations, dashed blue lines represent hydrogen bonds. For LigPlot+ visualizations, dashed green lines represent hydrogen bonds, half moons indicate van der Waals interactions, and red circles indicate that the residue has common interactions with other ligands in the figure.

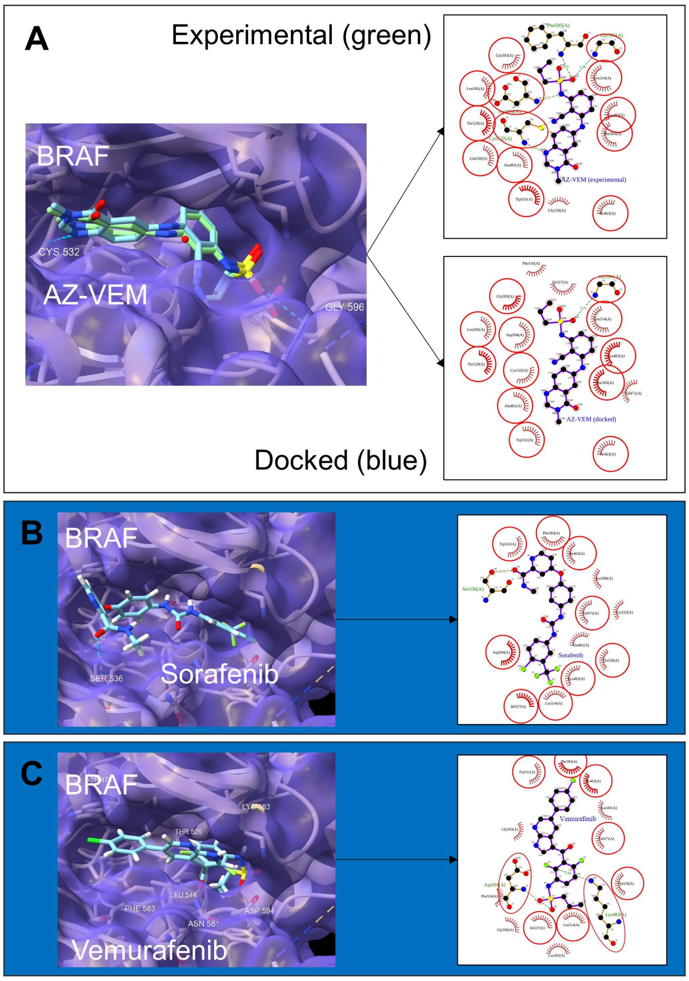

Figure S15. Docking results for wild-type BRAF (PDB code: 5ITA) with bound inhibitor AZ-VEM, sorafenib, and vemurafenib. **(A)** Comparison of experimental and docked pose of bound inhibitor AZ-VEM to wild-type BRAF as a positive docking control. Left: ChimeraX visualization of experimental pose (in green) overlaid with docked pose (in blue). Top right: LigPlot+ visualization of experimental pose. Bottom right: LigPlot+ visualization of docked pose. **(B)** Sorafenib docked to BRAF (SORA/VEM with probability = 1). **(C)** Vemurafenib docked to BRAF (SORA/VEM with probability = 1). For ChimeraX visualizations, dashed blue lines represent hydrogen bonds. For LigPlot+ visualizations, dashed green lines represent hydrogen bonds, half moons indicate van der Waals interactions, and red circles indicate that the residue has common interactions with other ligands in the figure.

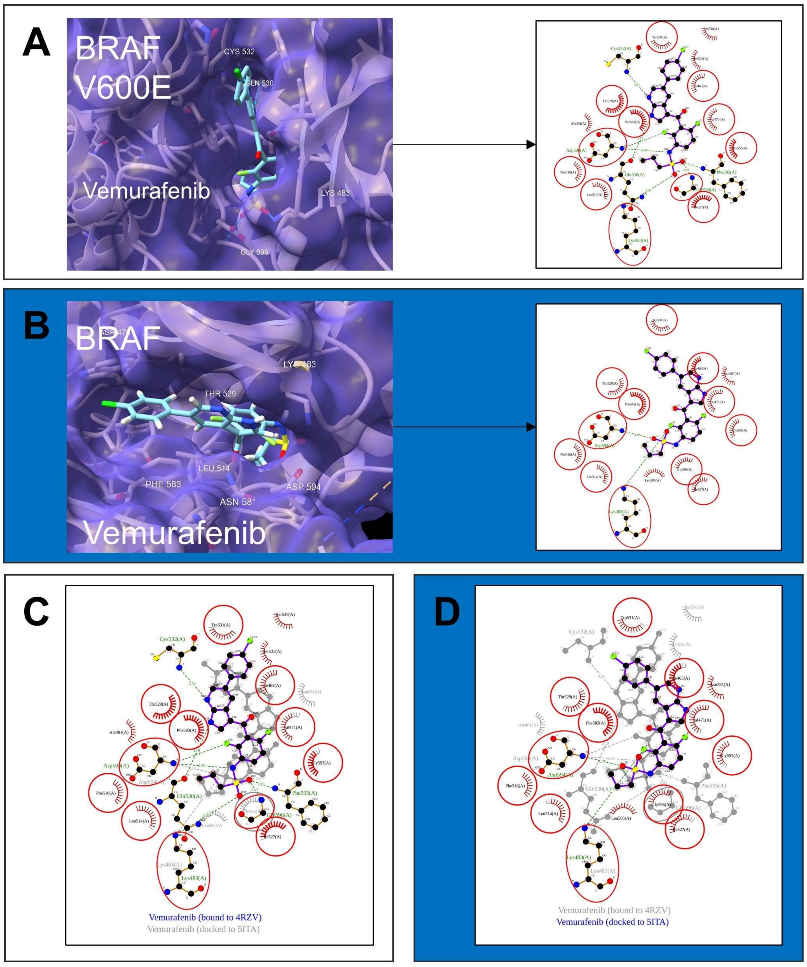

Figure S16. Comparison of vemurafenib bound to BRAF V600E (PDB code: 4RZV) and vemurafenib docked to wild type BRAF (PDB code: 5ITA). **(A)** vemurafenib bound to BRAF V600E (left: ChimeraX visualization, right: LigPlot+ visualization), **(B)** vemurafenib bound to wild type BRAF (left: ChimeraX, right: LigPlot+), **(C)** LigPlot+ visualization of vemurafenib bound to BRAF V600E superimposed onto vemurafenib docked to wild-type BRAF **(D)** LigPlot+ visualization of vemurafenib docked to wild-type BRAF superimposed onto vemurafenib bound to BRAF V600E. For ChimeraX visualizations, dashed blue lines represent hydrogen bonds. For LigPlot+ visualizations, dashed green lines represent hydrogen bonds, half moons indicate van der Waals interactions, and red circles indicate that the residue has common interactions with other ligands in the figure.

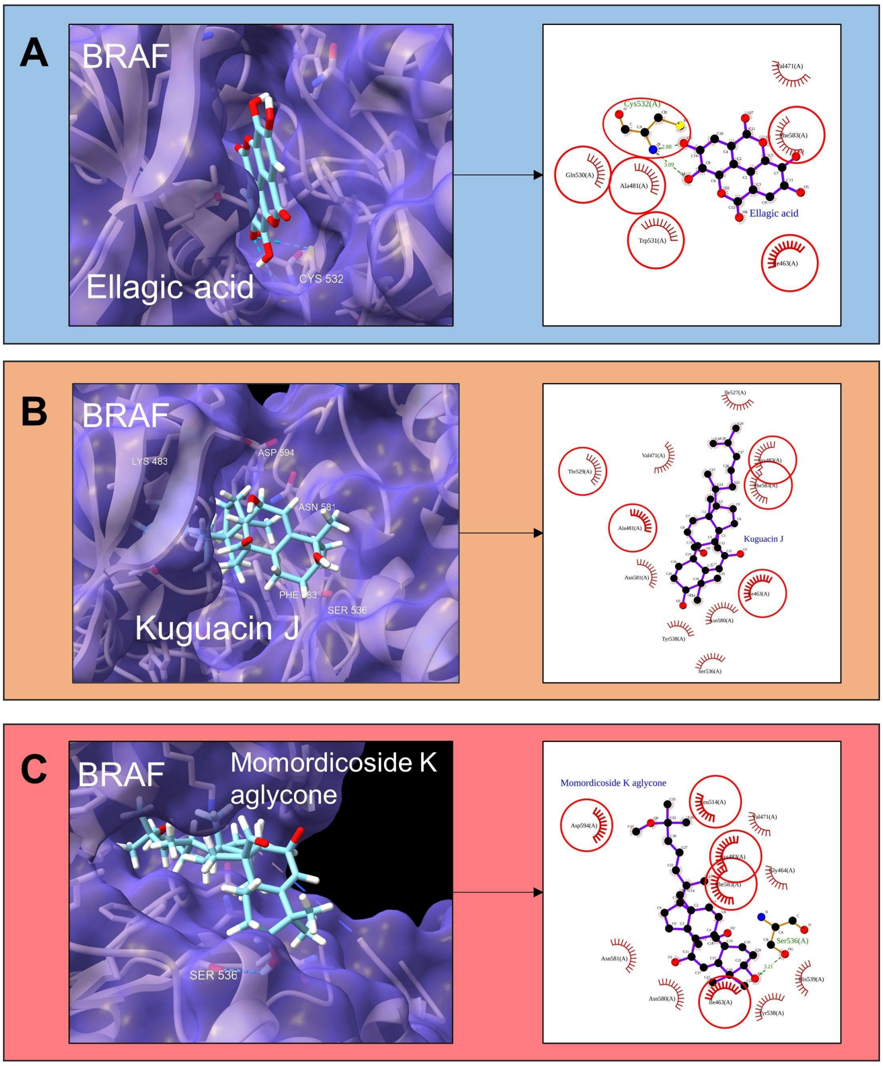

Figure S17. Visualizations of the three phytochemicals docked to BRAF (PDB code: 5ITA). Subfigure backgrounds are colored based on the docking confidence level of the interaction (see Figure S10 for detailed legend). Left panels: ChimeraX visualizations. Right panels: LigPlot+ visualizations. The phytochemical-protein pairs in each subfigure are as follows: **(A)** ellagic acid and BRAF (probability = 1), **(B)** kuguacin J and BRAF (medium docking confidence), and **(C)** momordicoside K aglycone and BRAF (low docking confidence). For ChimeraX visualizations, dashed blue lines represent hydrogen bonds. For LigPlot+ visualizations, dashed green lines represent hydrogen bonds, half moons indicate van der Waals interactions, and red circles indicate that the residue has common interactions with other ligands in the figure.

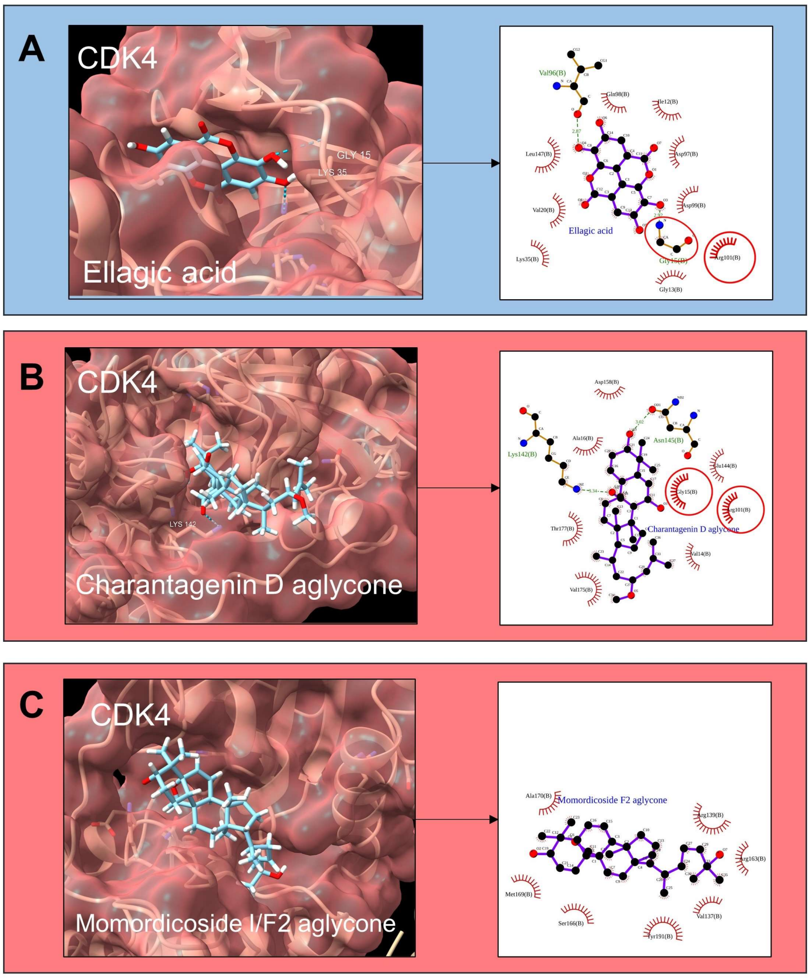

Figure S18. Visualizations of the three phytochemicals docked to CDK4 (PDB code: 2W96), specifically to the ATP-binding site of CDK4. Subfigure backgrounds are colored based on the docking confidence level of the interaction (see Figure S10 for detailed legend). Left panels: ChimeraX visualizations. Right panels: LigPlot+ visualizations. The phytochemical-protein pairs in each subfigure are as follows: **(A)** ellagic acid and CDK4 (probability = 1), **(B)** charantagenin D aglycone and CDK4 (low docking confidence), and **(C)** momordicoside I/F2 aglycone and CCND1 CDK4 (low docking confidence). For ChimeraX visualizations, dashed blue lines represent hydrogen bonds. For LigPlot+ visualizations, dashed green lines represent hydrogen bonds, half moons indicate van der Waals interactions, and red circles indicate that the residue has common interactions with other ligands in the figure.

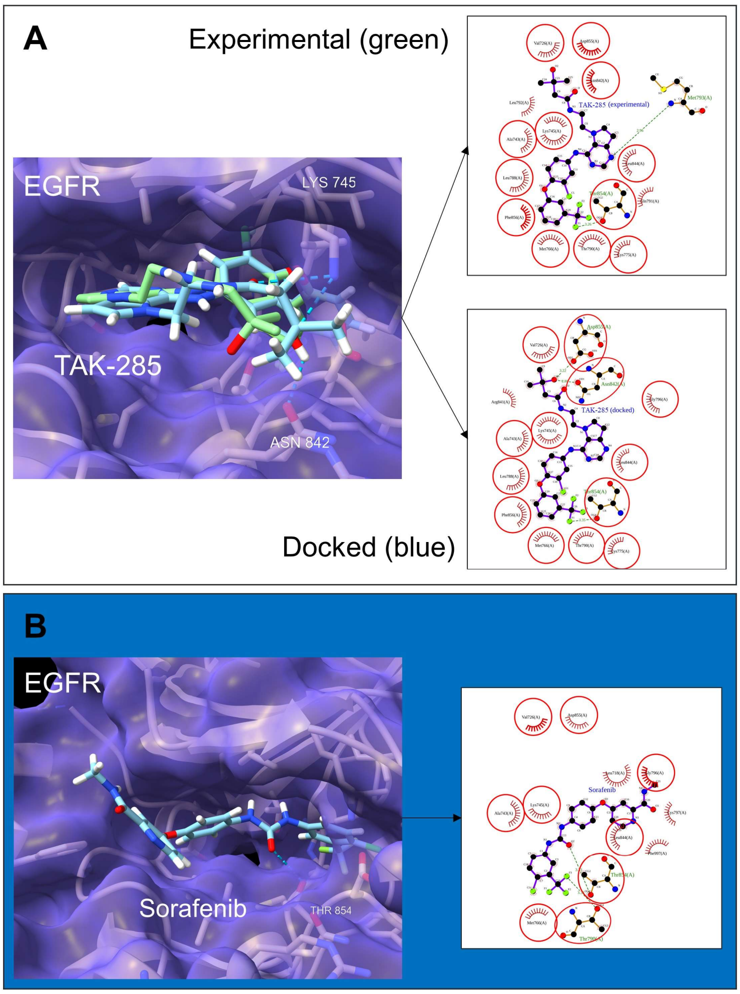

Figure S19. Docking results for EGFR (PDB code: 3POZ) with bound inhibitor TAK-285 and sorafenib. **(A)** Comparison of experimental and docked pose of bound inhibitor TAK-285 to EGFR as a positive docking control. Left: ChimeraX visualization of experimental pose (in green) overlaid with docked pose (in blue). Top right: LigPlot+ visualization of experimental pose. Bottom right: LigPlot+ visualization of docked pose. **(B)** Sorafenib docked to EGFR (SORA/VEM with probability = 1). For ChimeraX visualizations, dashed blue lines represent hydrogen bonds. For LigPlot+ visualizations, dashed green lines represent hydrogen bonds, half moons indicate van der Waals interactions, and red circles indicate that the residue has common interactions with other ligands in the figure.

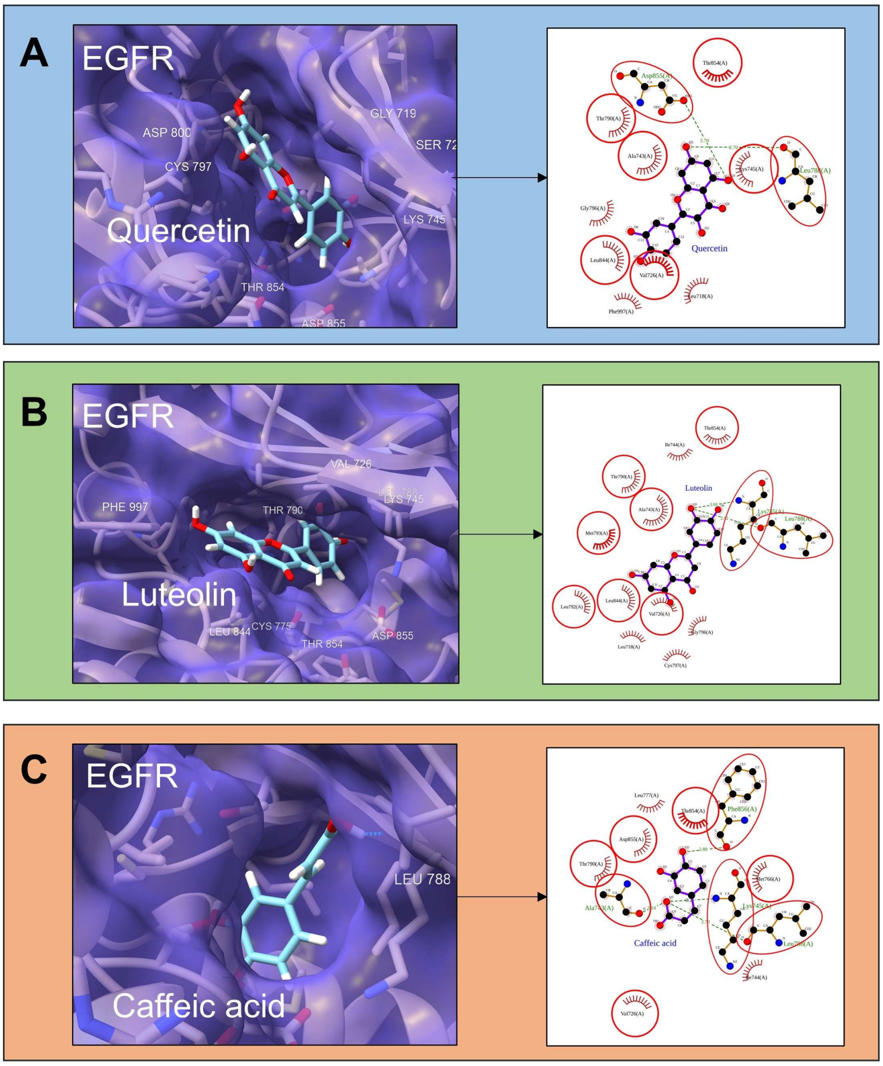

Figure S20. Visualizations of the three phytochemicals docked to EGFR (PDB code: 3POZ). Subfigure backgrounds are colored based on the docking confidence level of the interaction (see Figure S10 for detailed legend). Left panels: ChimeraX visualizations. Right panels: LigPlot+ visualizations. The phytochemical-protein pairs in each subfigure are as follows: **(A)** quercetin and EGFR (probability = 1), **(B)** luteolin and EGFR (high docking confidence), and **(C)** caffeic acid and EGFR (medium docking confidence). For ChimeraX visualizations, dashed blue lines represent hydrogen bonds. For LigPlot+ visualizations, dashed green lines represent hydrogen bonds, half moons indicate van der Waals interactions, and red circles indicate that the residue has common interactions with other ligands in the figure.

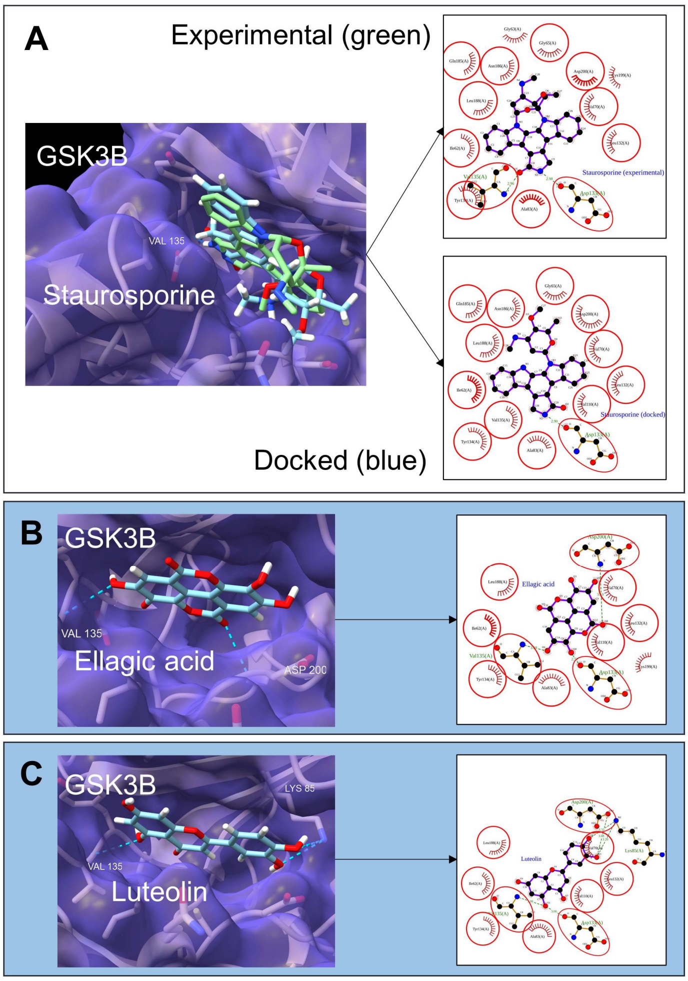

Figure S21. Visualizations of the three ligands docked to GS3KB (PDB code: 1Q3D). Subfigure backgrounds are colored based on the docking confidence level of the interaction (see Figure S10 for detailed legend). Left panels: ChimeraX visualizations. Right panels: LigPlot+ visualizations. The ligand-protein pairs in each subfigure are as follows: **(A)** Positive docking control: bound inhibitor staurosporine and GSK3B (Left panel: experimental pose in green, docked pose in blue. Right panels: experimental pose on top, docked pose on the bottom), **(B)** ellagic acid and GSK3B (probability = 1), and **(C)** luteolin and GSK3B (probability = 1). For ChimeraX visualizations, dashed blue lines represent hydrogen bonds. For LigPlot+ visualizations, dashed green lines represent hydrogen bonds, half moons indicate van der Waals interactions, and red circles indicate that the residue has common interactions with other ligands in the figure.

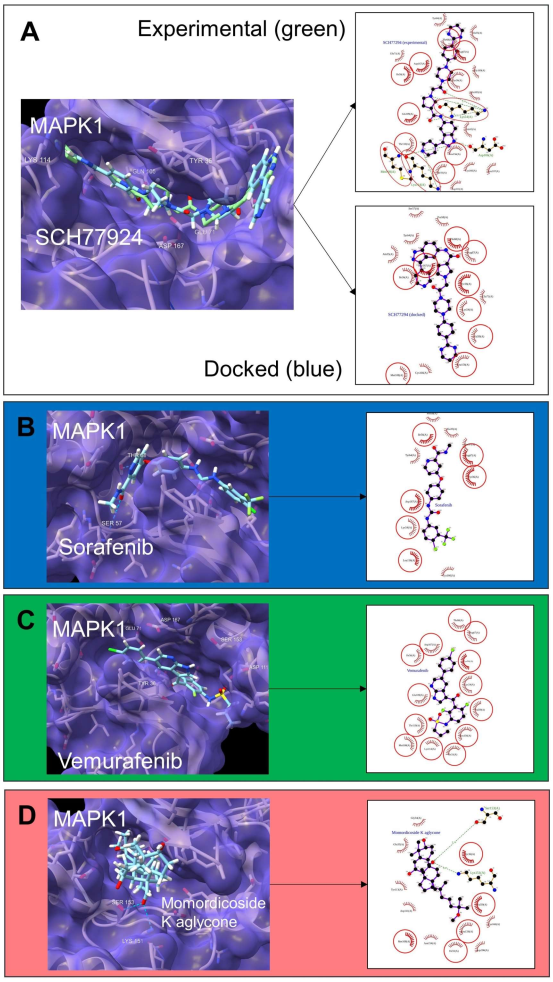

Figure S22. Visualizations of the four ligands docked to MAPK1 (PDB code: 4QTA). Subfigure backgrounds are colored based on the docking confidence level of the interaction (see Figure S10 for detailed legend). Left panels: ChimeraX visualizations. Right panels: LigPlot+ visualizations. **(A)** Positive docking control: bound inhibitor SCH77294 and MAPK1 (Left panel: experimental pose in green, docked pose in blue. Right panels: experimental pose on top, docked pose on the bottom). **(B)** Sorafenib docked to MAPK1 (SORA/VEM with probability = 1). **(C)** Vemurafenib docked to MAPK1 (SORA/VEM with high docking confidence). **(D)** Momordicoside K aglycone docked to MAPK1 (low docking confidence). For ChimeraX visualizations, dashed blue lines represent hydrogen bonds. For LigPlot+ visualizations, dashed green lines represent hydrogen bonds, half moons indicate van der Waals interactions, and red circles indicate that the residue has common interactions with other ligands in the figure.

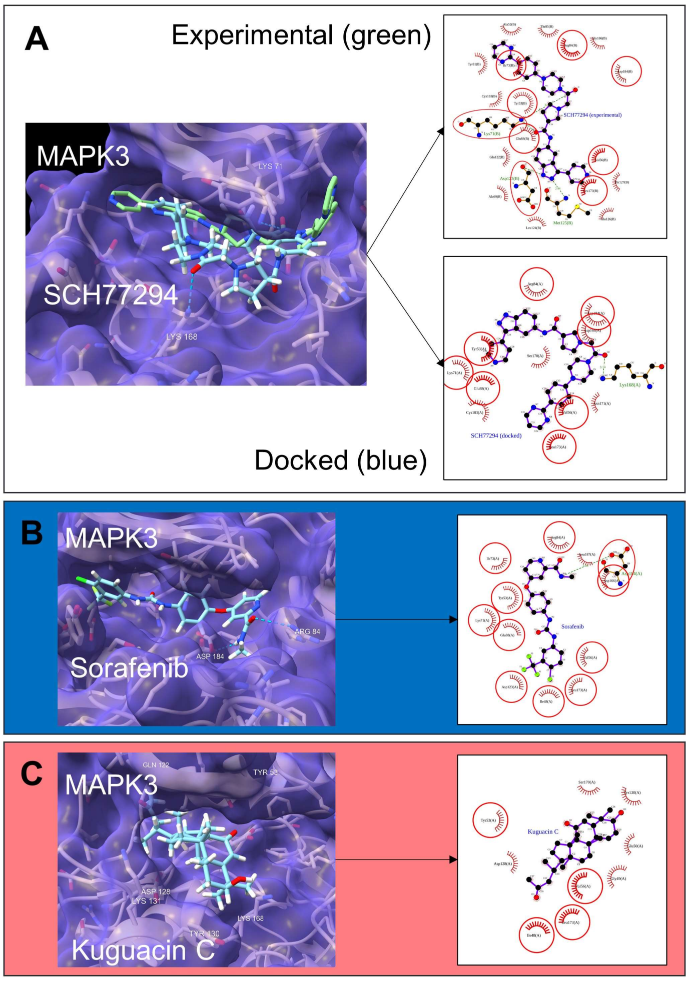

Figure S23. Visualizations of the three ligands docked to MAPK3 (PDB code: 4QTB). Subfigure backgrounds are colored based on the docking confidence level of the interaction (see Figure S10 for detailed legend). Left panels: ChimeraX visualizations. Right panels: LigPlot+ visualizations. **(A)** Positive docking control: bound inhibitor SCH77294 and MAPK3 (Left panel: experimental pose in green, docked pose in blue. Right panels: experimental pose on top, docked pose on the bottom). **(B)** Sorafenib docked to MAPK3 (SORA/VEM with probability = 1). **(C)** Kuguacin C docked to MAPK3 (low docking confidence). For ChimeraX visualizations, dashed blue lines represent hydrogen bonds. For LigPlot+ visualizations, dashed green lines represent hydrogen bonds, half moons indicate van der Waals interactions, and red circles indicate that the residue has common interactions with other ligands in the figure.

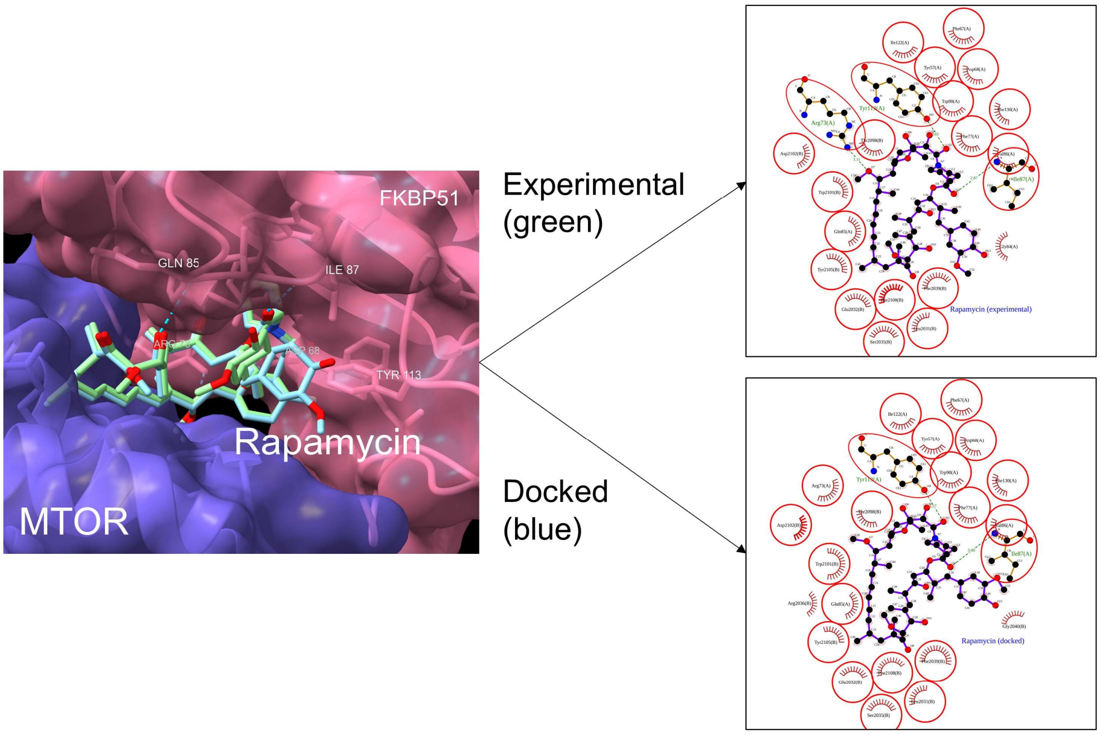

Figure S24. Comparison of experimental and docked pose of bound inhibitor rapamycin to MTOR (PDB code: 4DRI) as a positive docking control. Subfigure backgrounds are colored based on the docking confidence level of the interaction (see Figure S10 for detailed legend). Left: ChimeraX visualization of experimental pose (in green) overlaid with docked pose (in blue). Top right: LigPlot+ visualization of experimental pose. Bottom right: LigPlot+ visualization of docked pose. For ChimeraX visualizations, dashed blue lines represent hydrogen bonds. For LigPlot+ visualizations, dashed green lines represent hydrogen bonds, half moons indicate van der Waals interactions, and red circles indicate that the residue has common interactions with other ligands in the figure.

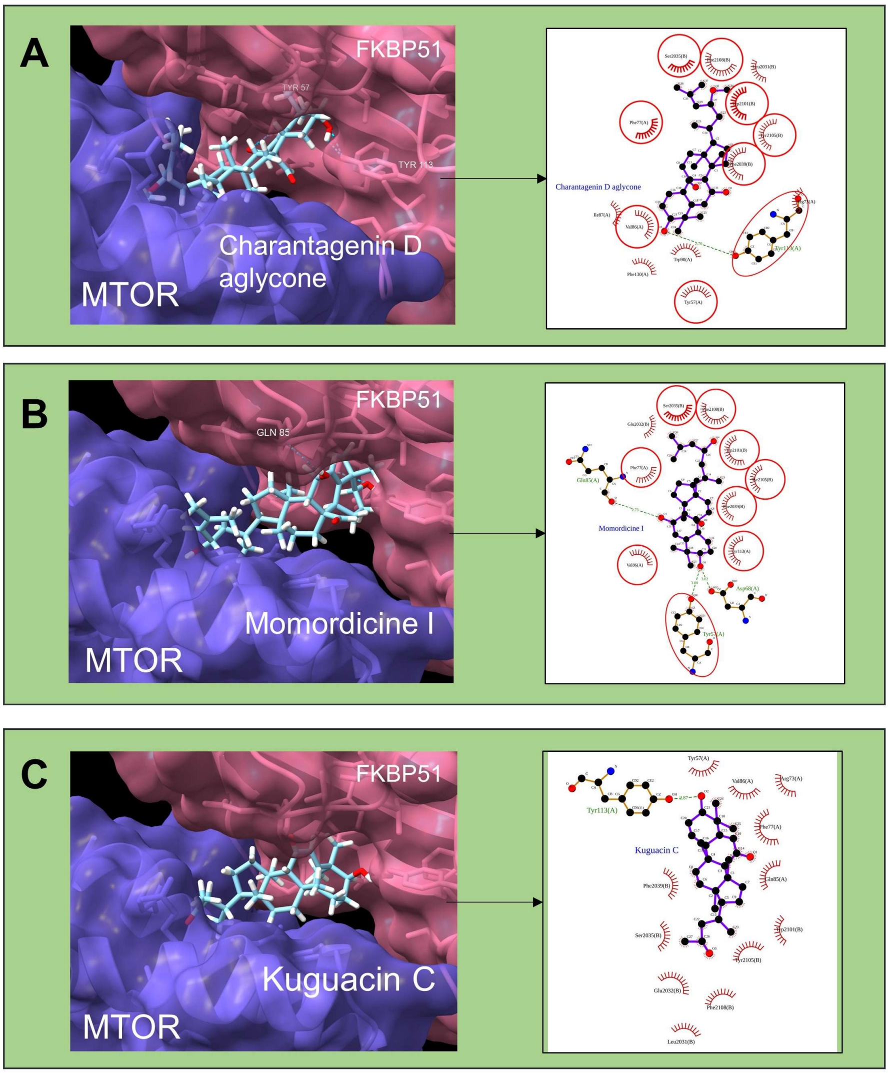

Figure S25. Visualizations of the three phytochemicals docked to MTOR (PDB code: 4DRI). Subfigure backgrounds are colored based on the docking confidence level of the interaction (see Figure S10 for detailed legend). Left panels: ChimeraX visualizations. Right panels: LigPlot+ visualizations. The phytochemical-protein pairs in each subfigure are as follows: **(A)** charantagenin D aglycone and MTOR (high docking confidence), **(B)** momordicine I and MTOR (high docking confidence), and **(C)** kuguacin C and MTOR (high docking confidence). For ChimeraX visualizations, dashed blue lines represent hydrogen bonds. For LigPlot+ visualizations, dashed green lines represent hydrogen bonds, half moons indicate van der Waals interactions, and red circles indicate that the residue has common interactions with other ligands in the figure.

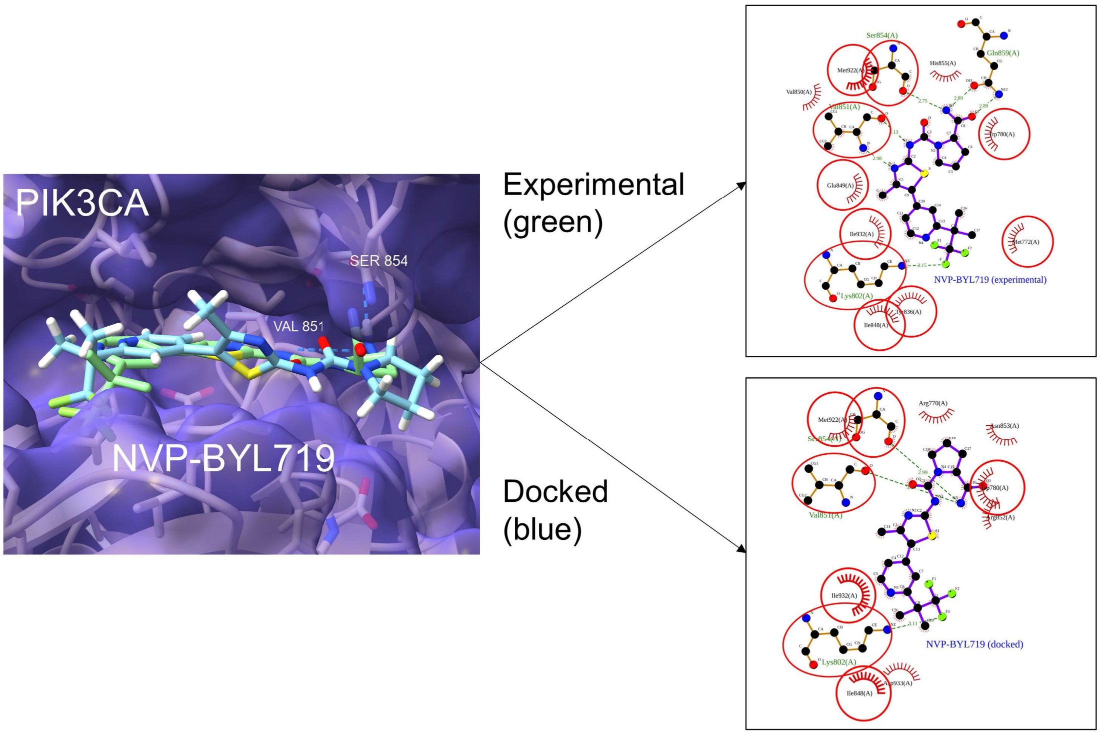

Figure S26. Comparison of experimental and docked pose of bound inhibitor NVP-BYL719 to PIK3CA (PDB code: 4JPS) as a positive docking control. Left: ChimeraX visualization of experimental pose (in green) overlaid with docked pose (in blue). Top right: LigPlot+ visualization of experimental pose. Bottom right: LigPlot+ visualization of docked pose. For ChimeraX visualizations, dashed blue lines represent hydrogen bonds. For LigPlot+ visualizations, dashed green lines represent hydrogen bonds, half moons indicate van der Waals interactions, and red circles indicate that the residue has common interactions with other ligands in the figure.

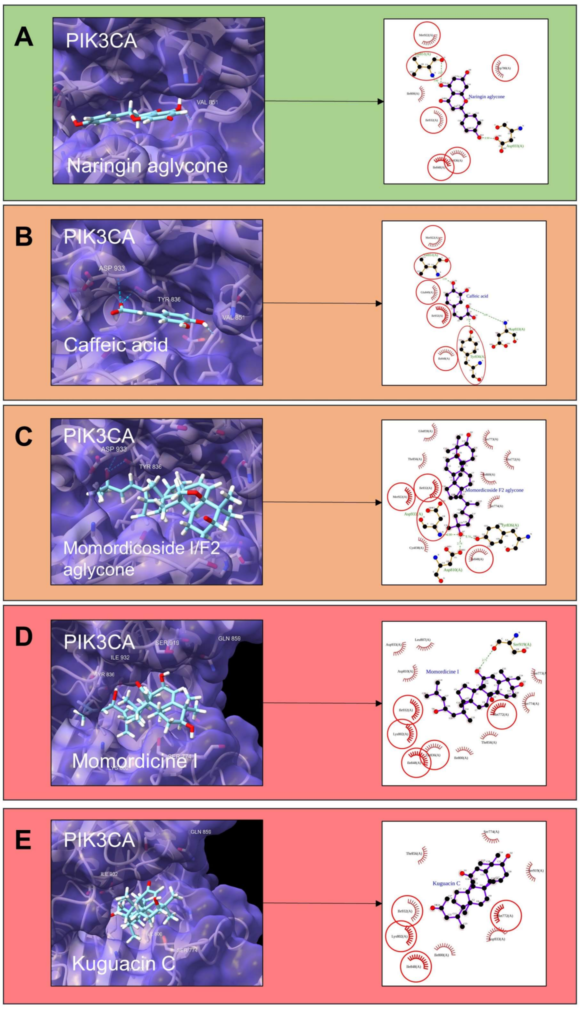

Figure S27. Visualizations of the five phytochemicals docked to PIK3CA (PDB code: 4JPS). Subfigure backgrounds are colored based on the docking confidence level of the interaction (see Figure S10 for detailed legend). Left panels: ChimeraX visualizations. Right panels: LigPlot+ visualizations. The phytochemical-protein pairs in each subfigure are as follows: **(A)** naringin aglycone and PIK3CA (high docking confidence), **(B)** caffeic acid and PIK3CA (medium docking confidence), **(C)** momordicoside I/F2 aglycone and PIK3CA (medium docking confidence), **(D)** momordicine I and PIK3CA (low docking confidence), and **(E)** kuguacin C and PIK3CA (low docking confidence). For ChimeraX visualizations, dashed blue lines represent hydrogen bonds. For LigPlot+ visualizations, dashed green lines represent hydrogen bonds, half moons indicate van der Waals interactions, and red circles indicate that the residue has common interactions with other ligands in the figure.

REFERENCES

Jia, S., Shen, M., Zhang, F., and Xie, J. (2017). Recent Advances in Momordica charantia: Functional Components and Biological Activities. *International Journal of Molecular Sciences* 18. doi:10.3390/ijms18122555

Mozaniel, S. d. O., Wanessa, A. d. C., Fernanda, W. F. B., Marilena, E. A., Gracialda, C. F., and Raul, N. d. C. J. (2018). Phytochemical profile and biological activities of Momordica charantia L. (Cucurbitaceae): A review. *African Journal of Biotechnology* 17, 829–846. doi:10.5897/AJB2017.16374

Raina, K., Kumar, D., and Agarwal, R. (2016). Promise of bitter melon (Momordica charantia) bioactives in cancer prevention and therapy. *Seminars in cancer biology* 40-41, 116–129. doi:10.1016/j.semcancer.2016.07.002
